# IGFBP‐3 and TGF‐β inhibit growth in epithelial cells by stimulating type V TGF‐β receptor (TβR‐V)‐mediated tumor suppressor signaling

**DOI:** 10.1096/fba.2021-00016

**Published:** 2021-06-16

**Authors:** Chun‐Lin Chen, Franklin W. Huang, Shuan Shian Huang, Jung San Huang

**Affiliations:** ^1^ Department of Biological Science National Sun Yat‐sen University Kaohsiung Taiwan; ^2^ Division of Hematology and Oncology Department of Medicine University of California San Francisco CA USA; ^3^ Auxagen Inc. St. Louis MO USA; ^4^ Departments of Biochemistry and Molecular Biology Saint Louis University School of Medicine St. Louis MO USA

**Keywords:** IGFBP‐3, IRS‐1/2, PP1_c_, PP2A_c_, TGF‐β, TβR‐V

## Abstract

The TGF‐β type V receptor (TβR‐V) mediates growth inhibition by IGFBP‐3 and TGF‐β in epithelial cells and loss of TβR‐V expression in these cells leads to development of carcinoma. The mechanisms by which TβR‐V mediates growth inhibition (tumor suppressor) signaling remain elusive. Previous studies revealed that IGFBP‐3 and TGF‐β inhibit growth in epithelial cells by stimulating TβR‐V‐mediated IRS‐1/2‐dependent activation and cytoplasm‐to‐nucleus translocation of IGFBP‐3‐ or TGF‐β‐stimulated protein phosphatase (PPase), resulting in dephosphorylation of pRb‐related proteins (p107, p130) or pRb, and growth arrest. To define the signaling, we characterized/identified the IGFBP‐3‐ and TGF‐β‐stimulated PPases in cell lysates and nucleus fractions in Mv1Lu cells treated with IGFBP‐3 and TGF‐β, using a cell‐free assay with ^32^P‐labeled casein as a substrate. Both IGFBP‐3‐ and TGF‐β‐stimulated PPase activities in cell lysates are abolished when cells are co‐treated with TGF‐β/IGFBP‐3 antagonist or RAP (LRP‐1/TβR‐V antagonist). However, the IGFBP‐3‐stimulated PPase activity, but not TGF‐β‐stimulated PPase activity, is sensitive to inhibition by okadaic acid (OA). In addition, OA or PP2A_c_ siRNA reverses IGFBP‐3 growth inhibition, but not TGF‐β growth inhibition, in Mv1Lu and 32D cells. These suggest that IGFBP‐3‐ and TGF‐β‐stimulated PPases are identical to PP2A and PP1, respectively. By Western blot/phosphorimager/immunofluorescence‐microscopy analyses, IGFBP‐3 and TGF‐β stimulate TβR‐V‐mediated IRS‐2‐dependent activation and cytoplasm‐to‐nucleus translocation of PP2A_c_ and PP1_c_, resulting in dephosphorylation of p130/p107 and pRb, respectively, and growth arrest. Small molecule TGF‐β enhancers, which potentiate TGF‐β growth inhibition by enhancing TβR‐I–TβR‐II‐mediated canonical signaling and thus activating TβR‐V‐mediated tumor suppressor signaling cascade (TβR‐V/IRS‐2/PP1/pRb), could be used to prevent and treat carcinoma.

**Abbreviations**
A549 cells: human Caucasian lung carcinoma cells
CDK: cyclin‐dependent kinase
CHO cells: Chinese hamster ovary epithelial cells
D32 cells: murine 32D myeloid cells
EMT: epithelial mesenchymal transition
IGF‐1: insulin‐like growth factor‐1
IGF‐2: insulin‐like growth factor‐1
IGFBP‐3: insulin‐like growth factor‐binding protein‐3
IR: insulin receptor
IRS‐1/2: insulin receptor substrate‐1/2
LRP‐1: low density lipoprotein receptor‐related protein‐1
Mv1Lu cells: mink lung epithelial cells
OA: okadaic acid
p107: p130, pRb‐related proteins
PAI‐1: plasminogen activator inhibitor‐1
PP1: protein phosphatase 1
PP1_c_
: 36‐kDa PP1 catalytic subunit
PP2A: protein phosphatase 2A
PP2A‐B56: a 56‐kDa substrate‐recognition B subunit of PP2A
PP2A_c_
: 37‐kDa PP2A catalytic subunit
PPase: protein phosphatase
pRb: retinoblastoma protein (p105)
RAP: receptor‐associated protein
siRNA: small interfering RNA
TGF‐β: transforming growth factor‐β
TβR‐I: type I TGF‐β receptor
TβR‐II: type II TGF‐β receptor
TβR‐III: type III TGF‐β receptor
TβR‐V: type V TGF‐β receptor
β_1_^25^
: TGF‐β peptide antagonist containing amino acid residues 41^st^ to 65^th^ of human TGF‐β_1_

## 1 INTRODUCTION

Insulin‐like growth factor‐binding protein‐3 (IGFBP‐3) is a growth regulator which exhibits IGF‐dependent and ‐independent growth inhibitory activities in target cells.^1^ In the IGF‐dependent activity, IGFBP‐3 inhibits cell growth by binding IGF‐1 and IGF‐2 and preventing them from binding to their receptor, the IGF‐1 receptor (IGF‐1R), in these cells. IGFBP‐3 is also capable of inhibiting growth of cells by directly interacting with its own specific receptor in cells. This specific IGFBP‐3 receptor in responsive cells has been identified as the type V TGF‐β receptor (TβR‐V) which was discovered in our lab in 1991.^2^, ^3^, ^4^, ^5^, ^6^, ^7^ It is identical to low density lipoprotein receptor‐related protein 1 (LRP‐1).^8^ IGFBP‐3 inhibits the growth of wild‐type mink lung epithelial cells (Mv1Lu cells), which express type I, type II, type III, and type V TGF‐β receptors (TβR‐I, TβR‐II, TβR‐III, and TβR‐V), TβR‐I‐deficient Mv1Lu cells (R1B cells), and TβR‐II‐deficient Mv1Lu cells (DR26 cells).^7^, ^9^, ^10^ Mv1Lu cells have been a model normal epithelial cell system to study TGF‐β activity and signaling.^7^ All of these wild‐type and mutant cells express TβR‐V. IGFBP‐3 does not bind to TβR‐I, TβR‐II, and TβR‐III in these cells.^4^, ^5^ The half maximal concentration of IGFBP‐3 for inhibiting growth of these cells is close to its K_d_ (0.3 µg/ml or 10 nM) for binding to TβR‐V,^4^, ^5^, ^7^ suggesting that IGFBP‐3‐induced growth inhibition is mainly mediated by TβR‐V in target cells. IGFBP‐3 maximally inhibits growth in these wild‐type and mutant cells by ~30%–60%.^4^, ^5^ The TβR‐V is absolutely required for growth inhibition by either IGFBP‐3 or TGF‐β in target normal epithelial cells.^2^, ^3^, ^4^, ^5^, ^6^, ^7^, ^9^, ^10^ IGFBP‐3 and TGF‐β are non‐covalent and covalent homodimers, respectively, containing a minimal active site motif of WS/CXD.^2^, ^3^, ^4^, ^11^, ^12^ They bind to the cell surface subdomains of TβR‐V at distinct sites. IGFBP‐3 and TGF‐β bind to cell surface subdomains II and IV, and a site between subdomains I and II of TβR‐V, respectively.^4^, ^5^, ^7^ TGF‐β at 50 pM mildly and moderately inhibits growth in cells expressing TβR‐V but lacking TβR‐I or TβR‐II such as R1B and DR26 cells by ~15 and ~30% growth inhibition, respectively.^9^, ^10^ However, TGF‐β at 1–5 pM potently inhibits growth (~100% inhibition) in wild‐type Mv1Lu cells by stimulating TβR‐V‐mediated growth inhibition (tumor suppressor) signaling in concert with canonical TGF‐β signaling (TβR‐I/TβR‐II/Smad2/3/4)^13^ in wild‐type Mv1Lu cells.^7^, ^9^ Canonical TGF‐β signaling potentiates TβR‐V‐mediated growth inhibition from 15 or 30% in mutant R1B and DR‐26 cells (at 50 pM TGF‐β) to ~100% TGF‐β (at 1–5 pM) growth inhibition by transcriptional activation of cyclin‐dependent kinase (CDK) inhibitors in wild‐type Mv1Lu cells.^14^ These suggest that TβR‐V mediates mild or moderate TGF‐β growth inhibition in mutant Mv1Lu cells (R1B and DR26 cells) lacking TβR‐I or TβR‐II, whereas TβR‐I–TβR‐II‐mediated canonical signaling is required for potent TGF‐β growth inhibition mediated by TβR‐V in wild‐type Mv1Lu cells. Absence of TGF‐β‐stimulated canonical signaling (TβR‐I/TβR‐II/Smad2/3/4) in R1B cells results in complete loss of TGF‐β (at ≤5 pM) growth inhibition activity in these cells.^9^

IGFBP‐3 and TGF‐β do not inhibit growth in cells lacking TβR‐V, such as homozygous LRP‐1‐deficient mouse embryonic fibroblasts (PEA‐13 cells), CHO cells deficient in LRP‐1 (CHO‐LRP‐1^−/−^ cells) and H1299 human non‐small cell lung carcinoma cells.^7^, ^10^ PEA‐13, H1299, and CHO‐LRP‐1^−/−^ cells express both TβR‐I and TβR‐II, and respond to TGF‐β‐stimulated TβR‐I/TβR‐II‐mediated transcriptional activation of extracellular matrix (ECM)‐related genes, such as PAI‐1.^10^ Wild‐type mouse embryonic fibroblasts (MEF) are sensitive to growth inhibition by either TGF‐β or IGFBP‐3.^10^ H1299 and CHO‐LRP‐1^−/−^ cells exhibit a spindle‐shaped fibroblastoid morphology, frequently observed in invasive carcinoma cells.^7^, ^10^, ^15^ Stable transfection of H1299 and CHO‐LRP‐1^−/−^ cells with TβR‐V/LRP‐1 cDNA confers sensitivity to either TGF‐β or IGFBP‐3 growth inhibition and restores normal squamous epithelial morphology.^10^, ^15^ These results suggest that TβR‐V is essential for IGF‐independent growth inhibition by IGFBP‐3 and potent growth inhibition by TGF‐β in epithelial cells. These results also support the notion that TβR‐V acts as a tumor suppressor gene which causes cancer when it is inactivated or turned off.^7^ This notion is also supported by the recent findings that primary tumors from a few hundred human patients with liver, colon and prostate cancers in China, France and Argentina, respectively, exhibit loss or very low levels of LRP‐1 (TβR‐V) expression.^16^, ^17^, ^18^ Understanding of the mechanisms whereby the TβR‐V mediates growth inhibition (tumor suppressor) signaling stimulated by IGFBP‐3 and TGF‐β should be important to elucidate the molecular basis of IGFBP‐3 and TGF‐β actions and to understand their roles in human cancers.^19^, ^20^ IGFBP‐3 and TGF‐β are moderate and potent growth inhibitory cytokines for epithelial cells, respectively. IGFBP‐3 acts as a tumor suppressor gene in several human carcinoma cancers examined.^20^ TGF‐β acts as a tumor suppressor at the early stage of carcinogenesis and a tumor promoter in late‐stage cancer.^19^ As a tumor suppressor, TGF‐β suppresses carcinogenesis by potently inhibiting growth in epithelial cells for maintaining normal squamous epithelial morphology and physiology.^21^

We previously demonstrated that IGFBP‐3 and TGF‐β inhibit growth in epithelial cells by stimulating TβR‐V‐mediated tumor suppressor signaling which involves IRS‐1/2‐dependent activation and cytoplasm‐to‐nucleus translocation of IGFBP‐3‐ or TGF‐β‐stimulated protein phosphatase (PPase), and dephosphorylation of retinoblastoma family proteins in the nucleus, resulting in cell growth arrest.^7^, ^10^, ^22^, ^23^ In this communication, we demonstrate the identification of IGFBP‐3‐ and TGF‐β‐stimulated PPases as PPase 2A (PP2A) and PPase 1 (PP1), which are the master regulators of the eukaryotic cell cycle, respectively, based on the distinct sensitivity of these PPase activities to okadeic acid (OA) and PP2A_c_ siRNA. By [Methy‐^3^H] thymidine incorporation/Western blot/phosphorimager/immunofluorescence‐microscopy analyses, we also demonstrate that IGFBP‐3 and TGF‐β stimulate IRS‐2‐dependent activation and cytoplasm‐to‐nucleus translocation of PP2A_c_ and PP1_c_, resulting in dephosphorylation of pRb‐related proteins (p130 or p107) and pRb (p105) in the nucleus, respectively, in epithelial cells and growth arrest.

## 2 MATERIALS AND METHODS

### 2.1 Materials

All chemicals used in the experiments were prepared as a 10 mM stock solution in DMSO. The final concentration of DMSO in all experiments was 0.1% or lower, which had no effect on IGFBP‐3 and TGF‐β activity. Human receptor‐associated protein (RAP) was provided by Dr. Dudley K. Strickland (Department of Vascular Biology, American Red Cross). [γ‐^32^P]ATP, [^32^P]‐orthophosphate and [methyl‐^3^H] thymidine (67 Ci/mmol) were purchased from ICN Biochemicals (Irvine, CA, USA). Okadaic acid (OA) was purchased from Tocris. IGFBP‐3 and TGF‐β1 (TGF‐β) were purchased from Peprotech. Insulin (A11382II) was purchased from Gibco. Primary antibodies against IRS‐1 (sc‐398), IRS‐2 (sc‐390761), PP1_c_ (37‐kDa catalytic subunit) (sc‐7482), pRb (p105) (sc‐65230), p107 (sc‐250), p130 (sc‐374521), phosphorylated Smad2 (P‐Smad2) (sc‐135644), Ser 270‐phosphorylated IRS‐1/2 (P‐IRS‐1/2) (sc‐17192), β‐actin (sc‐47778), and lamin B (sc‐6216) were purchased from Santa Cruz Biotechnology. Rabbit antibodies against N‐ and C‐terminal of human LRP‐1 (TβR‐V) were purchased from Sigma Chemical Co. and Abcam, respectively. Rabbit polyclonal antibodies against hyperphosphoryrated Rb (P‐Rb) (#8516) and PP2A_c_ (36‐kDa PP2A catalytic subunit) (#2038) were purchased from Cell Signaling Technology. Alexa Fluor 488‐ and 594‐conjugated secondary antibodies were purchased from Thermo Fisher. Secondary antibodies conjugated with horseradish peroxidase (Millipore, USA) and enhanced chemiluminescence (ECL) kit (Perkin‐Elmer Life Sciences) were used to develop immunoblots. TGF‐β peptide antagonist [β_1_^25^], a dual TGF‐β/IGFBP‐3 antagonist, was synthesized as previously described.^11^

### 2.2 Cell culture

Mv1Lu cells (CCL‐64) and human lung adenocarcinoma cell line A549 (CCL‐185) were purchased from ATCC. 32D cells (murine 32D myeloid cells stably expressing human insulin receptor (IR) and IRS‐2)^22^, ^23^ were provided by Dr. Martin G. Myers, Jr. (Joslin Diabetes Center, Harvard University). CHO‐K1 cells were purchased from American Type Culture Collection (Rockville, MD, USA). CHO‐LRP‐1^−/−^ cell^24^ were provided by Dr. Guejun Bu, Department of Pediatrics and Cell Biology and Physiology, Washington University School of Medicine. CHO‐LRP‐1^−/−^ cells were generated from CHO‐K1 cells by ethyl methane sulfate mutagenesis followed by pseudomonas exotoxin (PE)‐mediated selection of LRP‐1‐deficient cells.^24^ 32D cells stably expressing IR and IRS‐2 were grown in RPMI 1640 medium containing 10% fetal bovine serum and 5% WEHI conditioned medium according to the procedure provided by Dr. Martin G. Myers. CHO Cells were grown in DMEM/Ham's F‐12 medium containing 10% fetal bovine serum. Other cell lines used in this study were maintained in DMEM containing 50 U/mL each of penicillin and streptomycin and 10% fetal bovine serum (Invitrogen) in humidified incubators at 37°C and 5% CO_2_.

### 2.3 PPase activity assay

^32^P‐labeled casein was prepared by incubation of casein (21.6 mg) in 50 mM Tris–HCl, pH 7.0, containing 10% glycerol, 1 mM benzamidine, 0.1 mM PMSF, 14 mM mercaptoethanol, 0.2 mM [γ‐^32^P] ATP (200 cpm/pmol), 10 mM MgCl_2_, and 1.5 Unit/ml of the catalytic subunit of protein kinase A in a final volume of 3 ml. After overnight incubation at room temperature, the solution was filtered on a column (1.5 × 20 cm) of Sephadex 50G equilibriated in 50 mM Tris–HCl containing 10% glycerol and 1 mM benzamidine. Before stimulation with IGFBP‐3 or TGF‐β, cells were treated with or without 25 µg/ml of RAP (receptor‐associated protein) and 30 µg/ml of TGF‐β peptide antagonist (β_1_^25^) in serum‐free DMEM or DMEM/Ham's F‐12 medium for 10 min. The cells were stimulated with IGFBP‐3 (0.6 µg/ml) or TGF‐β (40 pM) for 3 hr. The cells were washed with cold phosphate‐buffered saline (PBS), detached with 50 mM Tris–HCl pH 7.0 containing 0.25 M sucrose, 5 mM EDTA, and pelleted at 1,500 rpm for 5 min at 4°C. The cells were then lysed in 50 µl of homogenization buffer (50 mM Tris–HCl, pH 7.0 containing 150 mM NaCl, 1% Triton X‐100, and 0.1 mM PMSF).

The PPase activity assay mixtures were composed of 50 mM Tris–HCl, pH 7.0 containing 10% glycerol, 1 mM benzamidine, 0.1 mM PMSF, 14 mM mercaptoethanol, 0.1 mg of bovine serum albumin (BSA), PPase‐containing sample (cell lysates or nucleus extracts containing 5 µg protein), and ^32^P‐labeled substrate in a final volume of 0.05 ml. Reactions in triplicates were initiated with the ^32^P‐labeled casein at 30°C, and after a 10 min reaction period, 0.1 ml of 10% trichloroacetic acid (TCA) was added. The mixture was centrifuged at 12,000 g for 2 min in a microcentrifuge. About 0.1 ml of the supernatant was then added to 1 ml scintillation counting liquid, and radioactivity was determined.

The lysates from cells treated with vehicle only exhibited non‐specific PPase activity (IGFBP‐3‐ or TGF‐β‐independent PPase activity with certain ~10^2^–10^3^ cpm; 200 cpm/pmol phosphate). This non‐specific PPase activity was subtracted from the total PPase activity in the cell lysates from cells treated with IGFBP‐3 or TGF‐β in order to estimate IGFBP‐3‐stimulated or TGF‐β‐stimulated PPase activity. For this reason, the mean (±SD) of the non‐specific PPase activity from triplicates was taken as 0 cpm in cells treated with vehicle only.

### 2.4 Immunofluorescence microscopy

One milliliter of culture media containing approximately 5,000–10,000 Mv1Lu cells was added to a 35 mm culture dish containing a square coverslip. Mv1Lu cells grown on coverslips were treated with IGFBP‐3 or TGF‐β. Cells were then fixed in 4% paraformaldehyde for 15 min followed by permeabilization. Fixed cells were blocked with 5% BSA in PBS for 20 min at room temperature (RT) and then incubated with an appropriate primary antibody solution overnight at 4°C. Fixed cells were incubated with Alexa Fluor‐conjugated secondary antibodies for 1 hr at RT. Samples were observed with a Zeiss AxioObserver Z1 microscope (Zeiss), and images were captured using AxioVision Rev 4.6 software. To determine the nuclear localization and the colocalization of PP1_c_ and hyperphosphorylated pRb (P‐Rb), the images were analyzed in three dimensions using an AxioObserver Z1 Apotome microscope (Zeiss). Colocalization was evaluated in single optical planes taken through the entire z‐axis of each cell. All images were acquired using identical intensity and photodetector gain to allow quantitative comparisons of relative levels of immunoreactivity between samples. All images were cropped and sized using ImageJ.

### 2.5 Nucleus fractionation for PPase activity assay

Nuclear extracts of the cells were prepared by hypotonic lysis followed by high salt extraction. Briefly, cell pellets were homogenized in 0.5 mL of ice‐cold lysis buffer, composed of 10 mM HEPES pH 7.9, 10 mM KCl, 2 mM MgCl_2_, 0.1 mM EDTA, 1 mM dithiothreitol (DTT), and 0.5 mM phenylmethylsulfonyl fluoride (all from Sigma Chemical Co.). The homogenates were centrifuged for 30 s at 500 g at 4°C to eliminate any unbroken tissue. The supernatants were incubated on ice for 20 min, vortexed for 30 s after the addition of 50 μL of 10% Nonidet P‐40 (Sigma Chemical Co.), and then centrifuged for 1 min at 5,000 g at 4°C. The crude nucleus pellet was suspended in 200 μL of ice‐cold extraction buffer (20 mM HEPES pH 7.9, 420 mM NaCl, 1.5 mM MgCl_2_, 0.1 mM EDTA, 1 mM DTT, and 0.5 mM PMSF) and incubated on ice for 30 min, mixed frequently, and centrifuged at 12,000 g at 4°C for 15 min. The supernatants were collected as nucleus extracts for Western blot and PPase activity assay. Protein concentration was determined using a bicinchoninic acid assay kit with BSA as the standard (Pierce Biochemicals).

### 2.6 siRNA interference

Murine PP2A_c_ siRNA oligonucleotide corresponding to nucleotide sequence 5’‐xxx‐3’ (ON‐TARGETplus SMARTpool Cat #: L‐040657–00) and negative control siRNA were obtained from Dharmacon. PP2A_c_ siRNA and negative control siRNA were resuspended in in RNase‐free water and stored at −80°C. Transfection of siRNA was carried out using electroporation (Bio‐Rad Gene Pulser Xcell Total System). Three million cells in 600 µl of RPMI 1640 were incubated with siRNA in a 0.4 cm cuvette for 5 min on ice before electroporation (260 V, 950 µF). After additional 5‐min incubation on ice, cells were re‐suspended in 12 ml of RPMI 1640 supplemented with glutamine and 10% FCS (fetal calf serum) without antibiotic. Antibiotics (1% penicillin/streptomycin) were added at 6 hr after electroporation. All measurements were performed at 24 or 72 hr after transfection.

### 2.7 [Methy‐^3^H] thymidine incorporation

Growth of OA‐treated Mv1Lu cells and PP2A_c_ siRNA knocked‐down 32D cells were determined by the measurement of [methy‐^3^H] thymidine incorporation into cellular DNA as described previously.^4^, ^9^, ^10^, ^12^ Briefly, cells grown to near confluence in 48‐well dishes were treated with several concentrations of OA at 37°C for 1 hr in serum‐free DMEM. The final concentration of DMSO was 0.2%. Treated cells were then incubated with 0.1 and 0.2 µg/ml IGFBP‐3, or 40 pM TGF‐β in DMEM containing 0.1% FCS at 37°C for 18 hr. The [methy‐^3^H] thymidine incorporation into cellular DNA was determined by incubation of cells with [methy‐^3^H] thymidine for 6 hr.

### 2.8 Western blot

Seventy‐two hours after siRNA transfection, 32D and Mv1Lu cells (3 × 10^6^ cells) were lysed with 100 µl, 50 mM Tris–HCl, pH 7.0 containing 1% Triton X‐100, 150 mM NaCl, 5 mM EDTA, and 0.1 mM PMSF. Cell lysates were subjected to 7.5% SDS‐PAGE and Western blotting using specific antibodies (Santa Cruz Biotechnology) as described previously.^22^, ^23^ The antigens on the blots were visualized using horseradish oxidase‐conjugated anti‐rabbit IgG antibody and ECL system.

### 2.9 Metabolic labeling and immunoprecipitation

Mv1Lu and 32D cells (3 × 10^6^ cells) grown in 6‐well plate were washed and incubated in phosphate‐free DMEM for 1 hr to deplete intracellular phosphate. After 2 hr of incubation with [^32^P] orthophosphate at 37°C in a CO_2_ incubator, cells were treated with 1 µg/ml of IGFBP‐3 and/or OA (and RAP) for 16 hr. Cell lysates were prepared by suspending cells in 600 µl of lysis buffer and p130 or p107 was immunoprecipitated with a rabbit polyclonal antibody against the N‐terminal domain of p130 or p107. The p130 or p107 antibody complex was captured with a protein G‐coated agarose beads. The immunoprecipitated proteins were resolved using 7.5% SDS‐PAGE. The gel was dried and autoradiographed by a phosphorimager.

### 2.10 Statistical analysis

Two‐tailed unpaired Student's *t*‐test was used for determining the significance of a difference between two (vehicle only and sample) means. It was mainly used to compare the means between two groups (vehicle only and one specific concentration of IGFBP‐3 or TGF‐β). The values were presented as mean ± SD. *p* < 0.05 was considered significant.

## 3 RESULTS

### 3.1 IGFBP‐3‐ and TGF‐β‐stimulated PPase activities are distinct in the sensitivity to OA inhibition in Mv1Lu cells

**FIGURE 1:**
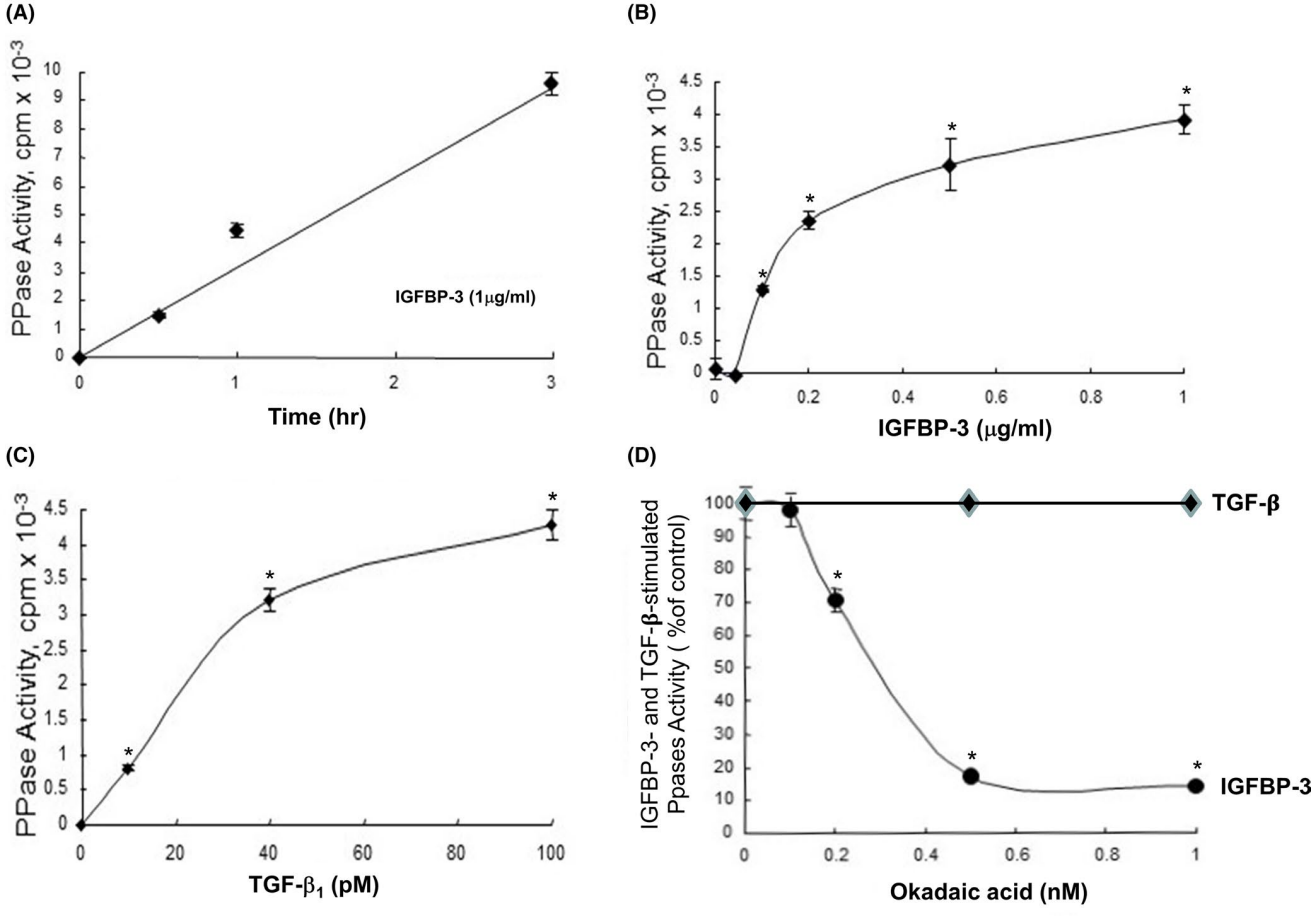
IGFBP‐3 and TGF‐β stimulate PPase activities in a time (A)‐ and concentration (B, C)‐dependent manner but with distinct sensitivity to OA inhibition (D) in Mv1Lu cells. Cells were treated with vehicle only, 1 µg/ml of IGFBP‐3 (A), different concentrations of IGFBP‐3 (B) and TGF‐β (C) or treated with 0.6 µg/ml of IGFBP‐3 or 40 pM TGF‐β in the presence of several concentrations of OA (D). After different time periods (A) or 2 hr incubation (B,C,D), cell lysates were assayed using ^32^P‐casein as substrate for assaying IGFBP‐3‐ or TGF‐β‐stimulated PPase activity. The IGFBP‐3‐ or TGF‐β‐stimulated PPase activity (200 cpm/pmole phosphate) was estimated by subtracting the ^32^P radioactivity (cpm) released from ^32^P‐casein by cell lysates of cells treated with vehicle only from that released by cell lysates of cells treated with IGFBP‐3 or TGF‐β. The assays were performed in triplicates. The data are mean ± SD *Significantly higher (B,C) or lower (D) than that of cells treated with vehicle only (control) or with IGFBP‐3 or TGF‐β only: *p *< 0.001

We previously proposed a model for the mechanisms by which IGFBP‐3 and TGF‐β inhibit growth in epithelial cells by stimulating TβR‐V/IRS‐1/2/PPase signaling.^7^ However, in this model, the identity of IGFBP‐3‐ or TGF‐β‐stimulated PPase was unknown. To characterize and identify the IGFBP‐3‐ and TGF‐β‐stimulated PPases, we developed a cell‐free PPase activity assay. In this assay, ^32^P‐phosphorylated casein, which was generated by ^32^P‐phosphate‐labeling (^32^P‐labeling) of casein (dephosphorylated) with protein kinase A in the presence of γ‐^32^P‐ATP, was incubated with cell lysates of Mv1Lu cells treated with or without IGFBP‐3 or TGF‐β1 (TGF‐β). After incubation, ^32^P‐phosphate released from ^32^P‐casein via the action of stimulated PPase in cell lysates and nucleus extracts were separated from remaining ^32^P‐casein by 10% trichloroacetic acid (TCA) precipitation in the presence of a carrier protein (BSA). The ^32^P‐phosphate released was recovered in the supernate of the 10% TCA solution. The IGFBP‐3‐ and TGF‐β‐stimulated PPase activities were estimated by subtracting the radioactivity of ^32^P‐phosphate released by cell lysates or nucleus extracts of cells treated without IGFBP‐3 or TGF‐β from that released by cell lysates or nucleus extracts of cells treated with IGFBP‐3 or TGF‐β. Using this assay, we characterized the kinetics, IGFBP‐3 or TGF‐β concentration dependence and OA sensitivity of the IGFBP‐3‐ or TGF‐β‐stimulated PPase activity in Mv1Lu cells. As shown in Figure 1, IGFBP‐3 and TGF‐β stimulated the PPase activities in a time‐ and concentration‐dependent manner. The IGFBP‐3‐stimulated PPase activity in the cell lysates appeared to be linear with time up to 3 hr treatment in these cells treated with 1 µg/ml of IGFBP‐3 (Figure 1A). The TGF‐β‐stimulated PPase activity in the cell lysates also exhibited a linear relationship with the treatment time for 3 hr in Mv1Lu cells treated with 40 pM TGF‐β (data not shown). IGFBP‐3 and TGF‐β stimulated the PPase activities in a concentration‐dependent manner (Figure 1B and Figure 1C, respectively). The half‐maximum concentration of IGFBP‐3 to stimulate the PPase activity was estimated to be ~10 nM (0.3 μg/ml) (Figure 1B) which is close to the half‐maximum concentration of IGFBP‐3 for binding to the IGFBP‐3 receptor (TβR‐V) and for inhibiting cell growth in Mv1Lu cells.^4^, ^5^ TGF‐β also stimulated a PPsse activity in Mv1Lu cells in a concentration‐dependent manner with a half‐maximum concentration of ~40 pM which is close to the K_d_ (50 pM) of TGF‐β binding to TβR‐V^2^, ^3^ in these cells (Figure 1C). TGF‐β at 10 pM stimulated a significant level of PPase activity (0.8 × 10^3^ cpm; 200 cpm/pmol phosphate) (Figure 1C). However, the IGFBP‐3‐stimulated PPase activity is distinct from the TGF‐β‐stimulated PPase activity in its greater sensitivity to OA inhibition. OA at 0.5 nM completely inhibited the IGFBP‐3‐stimulated PPase activity (Figure 1D). OA at 1 nM did not significantly affect the TGF‐β‐stimulated PPase activity (Figure 1D). These results suggest that IGFBP‐3 and TGF‐β stimulate PPase activities by interaction with TβR‐V in Mv1Lu cells and that IGFBP‐3‐ and TGF‐β‐stimulated PPases are different enzymes with distinct sensitivity to OA inhibition in these cells.

### 3.2 IGFBP‐3 and TGF‐β stimulate PPase activities in a TβR‐V‐dependent manner in Mv1Lu and CHO‐K1 cells

**FIGURE 2:**
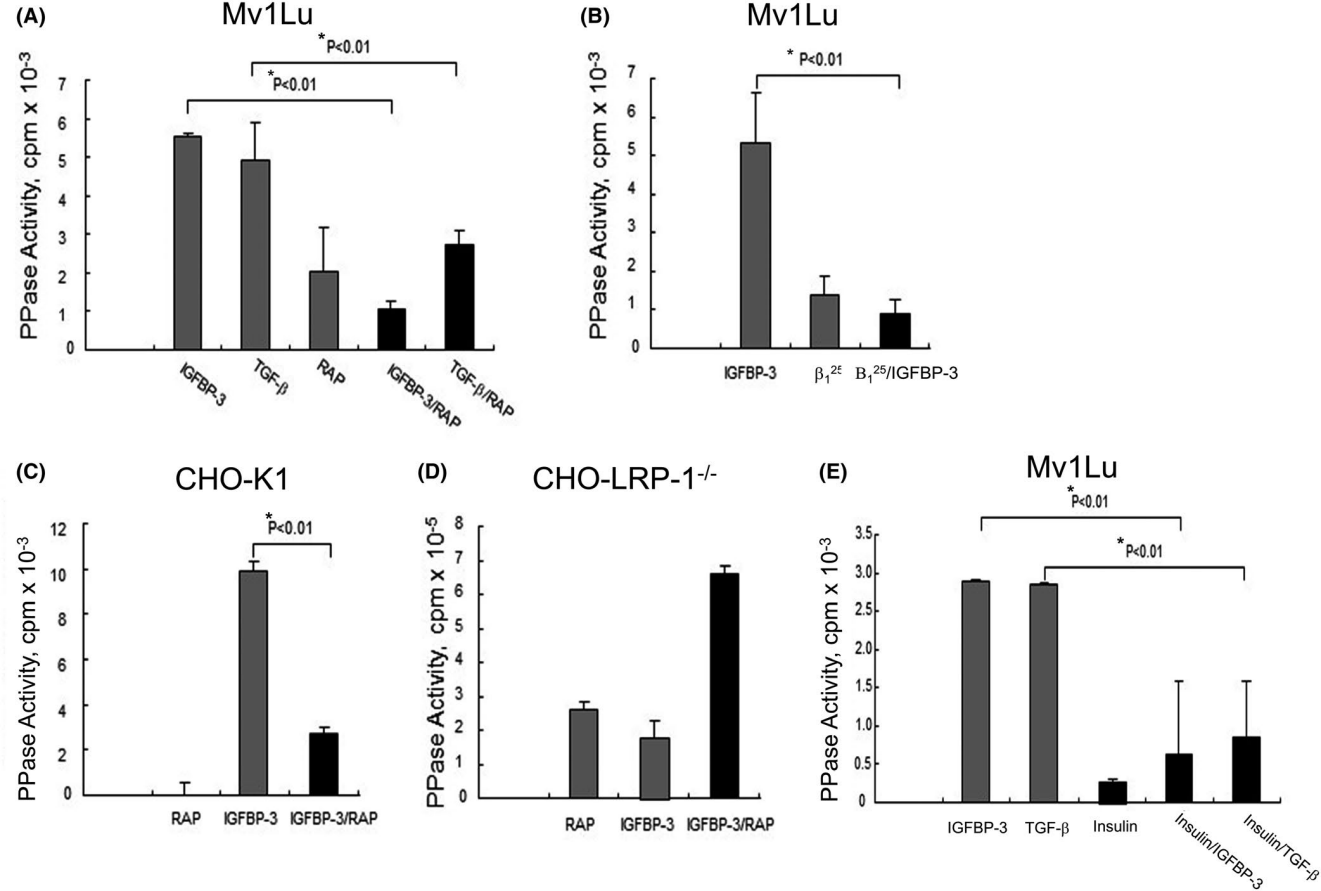
IGFBP‐3‐ and TGF‐β‐stimulated PPase activities are inhibited by co‐treating cells with LRP‐1 (TβR‐V) antagonist (RAP), TGF‐β peptide antagonist (β_1_^25^), or insulin in Mv1Lu (A, B, E) and CHO‐K1 (C) cells but not in CHO‐LRP‐1^−/−^ (D) cells. (A, B, E) Mv1Lu cells were treated with vehicle only, 1 µg/ml of IGFBP‐3 or 40 pM TGF‐β in the presence or absence of RAP (60 µg/ml), β_1_^25^ (10 µg/ml), or insulin (10 nM). After 2 hr at 37°C, cell lysates were assayed for IGFBP‐3‐ and TGF‐β‐stimulated PPase activities as described above. (C, D) Wild‐type CHO (CHO‐K1) (C) and CHO‐LRP‐1‐null (CHO‐LRP‐1^−/−^) (D) cells were treated with vehicle only or 1 µg/ml of IGFBP‐3 in the presence and absence of RAP (60 µg/ml). After 2 hr at 37°C, cell lysates were assayed for the IGFBP‐3‐ or TGF‐β‐stimulated PPase activity. The assays were performed in triplicates. The data are mean ± SD *Significantly lower than that of cells treated with IGFBP‐3 or TGF‐β only: *p*<0.01 (A, B, C, E). Both IGFBP‐3‐and RAP‐stimulated non‐specific (IGFBP‐3‐independent) PPase activity in CHO‐LRP‐1^−/−^ cells which lacked the expression of TβR‐V (LRP‐1) and did not exhibit TβR‐V‐mediated IGFBP‐3‐stimulated PPase activity (D). IGFBP‐3 and RAP exhibited the additive effects on stimulating non‐specific (IGFBP‐3‐independent) PPase activity (which could be mediated by different non‐specific PPases in cell lysates) in CHO‐LRP‐1^−/−^ cells. Combination of insulin with IGFBP‐3 or TGF‐β also exhibited additive effects on stimulating non‐specific (IGFBP‐3‐ or TGFβ‐independent) PPase activity in Mv1Lu cells (E)

The TβR‐V has been identified as the IGFBP‐3 receptor which mediates its IGF‐independent growth inhibitory activity.^2^, ^3^, ^4^, ^5^ It has also been identified as an important TGF‐β receptor required for mediating TGF‐β growth inhibitory activity when canonical signaling mediated by TβR‐I and TβR‐II potentiates TGF‐β growth inhibitory activity (~100% at 1–5 pM TGF‐β) in wild‐type Mv1Lu cells (7.9.10). To define the role of TβR‐V in mediating IGFBP‐3‐ and TGF‐β‐stimulated PPase activities, we determined the effects of TβR‐V antagonists such as a dual TGF‐β/IGFBP‐3 peptide antagonist (β_1_^25^)^4^, ^11^ and RAP, a LRP‐1 (TβR‐V) antagonist,^25^ and 10 nM insulin^22^, ^23^ on IGFBP‐3‐ and TGF‐β‐stimulated PPase activities in Mv1Lu cells, and wild‐type and LRP‐1 (TβR‐V)‐deficient Chinese hamster ovary (CHO) epithelial cells (CHO‐K1 and CHO‐LRP‐1^−/−^ cells, respectively). As shown in Figure 2, IGFBP‐3‐ and TGF‐β‐stimulated PPase activities in Mv1Lu cells (Figure 2A,B,E) and wild‐type CHO cells (CHO‐K1 cells) (Figure 2C). Treatment of these cells with RAP or β_1_^25^ alone stimulated non‐specific (IGFBP‐3‐ or TGF‐β‐independent) PPase activity (Figure 2A,B). Co‐treatment of these cells with RAP or β_1_^25^ and IGFBP‐3 or TGF‐β stimulated non‐specific (IGFBP‐3‐ or TGF‐β‐independent) PPase activity which was statistically indifferent from that treated with RAP or β_1_^25^ alone, suggesting that RAP or β_1_^25^ completely abolished the IGFBP‐3‐ and TGF‐β‐stimulated PPase activities (Figure 2A,B, respectively). The important role of TβR‐V in mediating the IGFBP‐3‐stimulated PPase activity was further supported by experiments using CHO‐K1 and CHO‐LRP‐1^−/−^ cells (Figure 2C,D, respectively). IGFBP‐3 stimulated a PPase activity which was blocked in the presence of RAP in CHO‐K1 cells (Figure 2C). In Figure 2C, the RAP alone value was taken as 0 cpm. It means that RAP alone, like vehicle only, exhibited non‐specific (IGFBP‐3‐independent) PPase activity which was taken as a mean of 0 cpm (±SD, n = 3). In Figure 2D, both IGFBP‐3‐ and RAP‐stimulated non‐specific (IGFBP‐3‐independent) PPase activity in CHO‐LRP‐1^−/−^ cells which lacked the expression of LRP‐1 (TβR‐V) and did not exhibit IGFBP‐3‐stimulated PPase activity. IGFBP‐3 and RAP appeared to exert additive effects on stimulating non‐specific (IGFBP‐3‐independent) PPase activity in CHO‐LRP‐1^−/−^ cells (Figure 2D). However, deficiency of LRP‐1 appeared to greatly increase non‐specific (IGFBP‐3‐independent) PPase activity up to ~10^5^ cpm activity baseline levels in these CHO‐LRP‐1^−/−^ cells as compared to those (10^3^ cpm activity baseline levels) seen in wild‐type CHO‐K1 cells (Figure 2D vs. Figure 2C). LRP‐1 (TβR‐V) acts as a tumor suppressor for epithelial cells.^7^ Loss of LRP‐1 in CHO‐K1 cells leads to transformation into carcinoma cells (CHO‐LRP‐1^−/−^ cells) which exhibited a spindle‐shaped fibroblastoid morphology, frequently observed in invasive carcinoma cells.^15^ Transformation of wild‐type CHO epithelial cells into CHO‐LRP‐1^−/−^ cells (carcinoma cells) appeared to greatly upregulate non‐specific (IGFBP‐3‐independent) PPase activity in these CHO‐LRP‐1^−/−^ cells.

We previously demonstrated that insulin at 10 nM blocks IGFBP‐3‐induced growth inhibition in Mv1Lu cells^22^ and partially blocks TGF‐β‐induced growth inhibition in the presence of anti‐α5β1 integrin in these cells.^23^ We also demonstrated that insulin (10 nM)‐activated IGF‐1R catalyzes tyrosine‐phosphorylation of IRS‐1/2, conferring resistance of tyrosine‐phosphorylated IRS‐1/2 to IGFBP‐3‐stimulated dephosphorylation and IGFBP‐3‐induced growth inhibition in these cells.^22^ We hypothesize that IGFBP‐3‐ and TGF‐β‐stimulated PPases are involved in IGFBP‐3‐ and TGF‐β‐induced growth inhibition, respectively, in target cells.^7^ We examined the effects of insulin on IGFBP‐3‐ and TGF‐β‐stimulated PPase activities. Mv1Lu cells were treated with insulin (10 nM) and IGFBP‐3 (0.3 µg/ml) or TGF‐β (50 pM) simultaneously for 2 hr. It is important to note that IGF‐1R‐catalyzed tyrosine phosphorylation of IRS‐1/2 occurs much faster than PPase‐catalyzed dephosphorylation of these proteins.^22^, ^23^ The IGFBP‐3‐ or TGF‐β‐stimulated PPase activity in the cell lysates of stimulated cells was determined using ^32^P‐phosphorylated casein as a substrate. As shown in Figure 2E, insulin (10 nM), effectively attenuated IGFBP‐3‐ and TGF‐β‐stimulated PPase activities in Mv1Lu cells. This is consistent with the notion that insulin‐stimulated IGF‐1R‐catalyzed tyrosine phosphorylation of IRS‐1/2 results in the inability of the tyrosine‐phosphorylated IRS‐1/2 to mediate activation of either IGFBP‐3‐stimulated PPase or TGF‐β‐stimulated PPase in these cells treated with IGFBP‐3 or TGF‐β.^22^, ^23^ Combination of insulin with IGFBP‐3 or TGF‐β appeared to have additive effects on stimulating non‐specific (IGFBP‐3‐independent) PPase activity (in Figure 2E). These results support the notion that non‐tyrosine‐phosphorylated but Ser/Thr‐phosphorylated IRS‐1/2 are involved in TβR‐V‐mediated activation of IGFBP‐3‐ and TGF‐β‐stimulated PPases, and in TβR‐V‐mediated cell growth inhibition by IGFBP‐3 and TGF‐β.^10^, ^22^, ^23^

### 3.3 IGFBP‐3 and TGF‐β stimulate colocalization of TβR‐V and IRS‐1/2 at the plasma membrane and cytoplasm‐to‐nucleus translocation of IRS‐2 and IGFBP‐3‐stimulated PPase complexes in Mv1Lu cells

**FIGURE 3:**
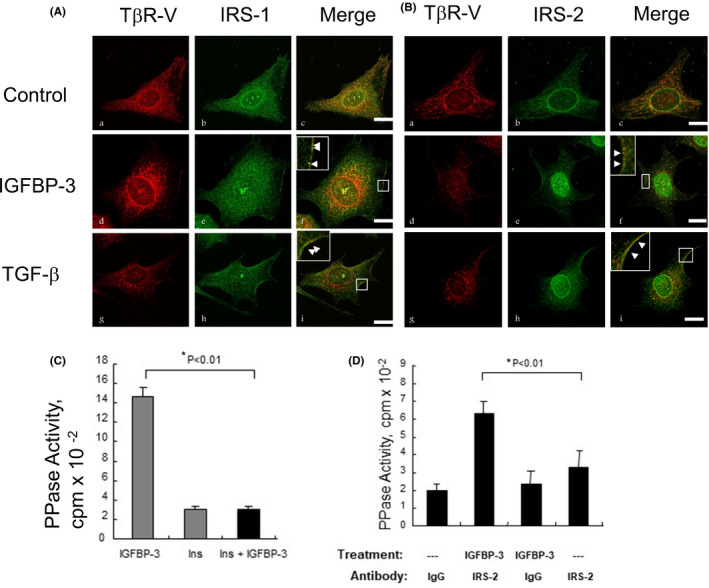
IGFBP‐3 or TGF‐β stimulates co‐localization of TβR‐V and IRS‐1 (A) or IRS‐2 (B) at the plasma membrane and cytoplasm‐to‐nucleus translocation of IRS‐2 (B), but not IRS‐1 (A), and cytoplasm (C)‐to‐nucleus (D) translocation of IRS‐2‐associated IGFBP‐3‐stimulated PPase activity in Mv1Lu cells. (A, B) Cells grown on coverslips in p35 culture dishes were treated with control (vehicle only), IGFBP‐3 (1 µg/ml) or TGF‐β (40 pM). K_d_ for IGFBP‐3 and TGF‐β binding to TβR‐V were estimated to be 10 nM (0.3 µg/ml) and 50 pM, respectively.^3^, ^4^ After 2 hr at 37°C, cells were fixed and stained by immunofluorescence using antibodies to TβR‐V and IRS‐1/2. Arrowheads indicate the co‐localization of TβR‐V and IRS‐1/2 at the plasma membrane (Af,i and Bf,i, inset); scale bar = 10 microns. Both IGFBP‐3‐ and TGF‐β‐stimulated cytoplasm‐to‐nucleus translocation of IRS‐2 but not IRS‐1 (Be,h vs. Ae,h). (C,D) Cells were treated with IGFBP‐3 (1 µg/ml), insulin (10 nM), or insulin (10 nM) + IGFBP‐3 (1 µg/ml) for cell lysate assay, and with IGFBP‐3 (1 µg/ml) and vehicle only (‐‐‐) for nucleus extract assay. After 2 hr at 37°C, cell lysates (C) were assayed for IGFBP‐3‐stimulated PPase activity and nucleus extracts (D) were immunoprecipitated with control IgG and IgG to IRS‐2. The immunoprecipitates were then assayed for the IGFBP‐3‐stimulated PPase activity. The assays were performed in triplicates. The data are mean ± SD *Significantly lower (C) or higher (D) than that of cells treated with IGFBP‐3 only or vehicle only: *p *< 0.01

We previously hypothesized that IGFBP‐3 and TGF‐β induce growth inhibition by interaction with TβR‐V, which recruits IRS‐1/2 and IGFBP‐3‐stimulated or TGF‐β‐stimulated PPase to form ternary complexes at the cytoplasmic tail of TβR‐V, resulting in the activation of IGFBP‐3‐stimulated PPase or TGF‐β‐stimulated PPase, dephosphorylation of IRS‐1/2 by activated IGFBP‐3‐ or TGF‐β‐stimulated PPase in the ternary complexes at the cytoplasmic tail of TβR‐V, dissociation of dephosphorylated IRS‐1/2‐IGFBP‐3‐stimulated PPase or dephosphorylated IRS‐1/2‐TGF‐β‐stimulated PPase binary complexes from the cytoplasmic tail of TβR‐V, and subsequent translocation of dephosphorylated IRS‐1/2‐IGFBP‐3‐stimulated PPase or dephosphorylated IRS‐1/2‐TGF‐β‐stimulated PPase binary complexes from cytoplasm to the nucleus where it induces cell cycle arrest by dephosphorylating retinoblastoma‐family proteins.^7^ To test this hypothesis, we performed immunofluorescence microscopy of Mv1Lu cells treated with and without (0.3 µg/ml) IGFBP‐3 and TGF‐β using specific antibodies to TβR‐V/LRP‐1 and IRS‐1/2. As shown in Figure 3A,B, IGFBP‐3 and TGF‐β‐stimulated colocalization of TβR‐V and IRS‐1/2 at the plasma membrane (Figure 3Af,i and Figure 3Bf,i, inset) and cytoplasm‐to‐nucleus translocation of IRS‐2 but not IRS‐1 (Figure 3Be,h vs. Figure 3Ae,h). These results suggest that IGFBP‐3 and TGF‐β stimulate complex formation of TβR‐V, IRS‐1/2, and possibly IGFBP‐3‐ or TGF‐β‐stimulated PPase at the plasma membrane and cytoplasm‐to‐nucleus translocation of IRS‐2 likely as PPase complexes in Mv1Lu cells. To analyze the presence of IGFBP‐3‐stimulated PPase‐IRS‐2 complexes in nucleus extracts of Mv1Lu cells, the IGFBP‐3‐stimulated PPase activity associated with IRS‐2 in nucleus extracts was then determined. As shown in Figure 3C, IGFBP‐3 stimulated a PPase activity in cell lysates of treated Mv1Lu cells. Approximately 50% of it was present in nucleus extracts and could be immunoprecipitated by antibodies to IRS‐2 (Figure 3D vs. Figure 3C). Insulin completely abolished the IGFBP‐3‐stimulated PPase activity in cell lysates (Figure 3C). These results suggest that IGFBP‐3 stimulates complex formation and cytoplasm‐to‐nucleus translocation of IGFBP‐3‐stimulated PPase and IRS‐2 in Mv1Lu cells.

### 3.4 IGFBP‐3‐induced growth inhibition, but not TGF‐β‐induced growth inhibition, is reversed by OA and PP2A_c_ siRNA in Mv1Lu and 32D cells

**FIGURE 4:**
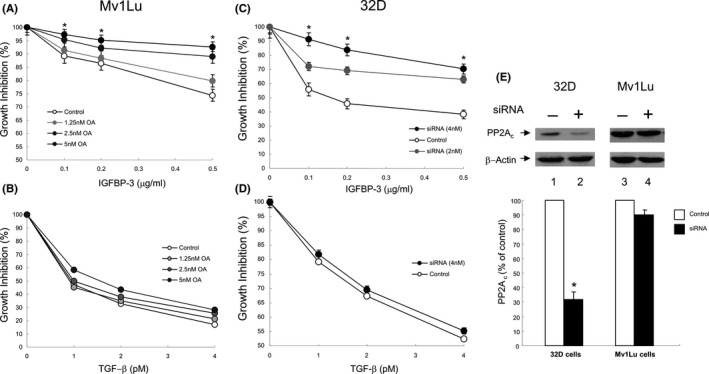
IGFBP‐3‐ but not TGF‐β‐induced growth inhibition is reversed by OA in Mv1Lu cells (A, B) and by PP2A_c_ siRNA in 32D cells (C, D) via attenuating PP2A_c_ expression in these cells (E). (A,B) Mv1Lu cells were treated with several concentrations of OA for 1 hr at 37°C and then treated with several concentrations IGFBP‐3 (A) or TGF‐β (B). After 18 hr at 37°C, the cell growth was determined by measurement of [methy‐^3^H] thymidine incorporation into cellular DNA. The [methy‐^3^H] thymidine incorporation in cells incubated without OA was taken as 100% cell growth. The reversibility (%) by OA of IGFBP‐3‐ or TGF‐β‐induced growth inhibition was estimated by 100 × (IGFBP‐3‐ or TGF‐β‐induced growth inhibition in the absence of OA – IGFBP‐3‐ or TGF‐β‐induced growth inhibition in the presence of OA)/IGFBP‐3‐ or TGF‐β‐induced growth inhibition in the absence of OA). The assays were performed in triplicates. The data are mean ± SD *Significantly lower than that of cells treated with control (vehicle only) (A): *p *< 0.01. (C,D)​ 32D cells were transfected with 0, 2, and 4 nM (C) or 0 and 4 nM (D) murine PP2A_c_ siRNA by electroporation. Transfected cells were treated with several concentrations of IGFBP‐3 (C) or TGF‐β (D). After 18 hr at 37°C, the cell growth was determined by measurement of [methy‐^3^H] thymidine incorporation into cellular DNA. The cell growth in cells treated with control siRNA (control) was taken as 100% (C, D). The data are mean ± SD *Significantly lower than that of cells treated with control siRNA (control) (C): *p* < 0.01. (E) Murine PP2A_c_ siRNA attenuates the expression of PP2A_c_ protein in murine 32D cells but not in mink Mv1Lu cells. 32D and Mv1Lu cells were transfected with control siRNA (−) and 4 nM murine PP2A_c_ siRNA (+) by electroporation. Transfected cells were analyzed by Western blot analysis using antibody to PP2A_c_ (top panel), which were representatives of a total of three experiments, and quantified by densitometry (bottom panel). Murine PP2A_c_ siRNA was effective in attenuating PP2A_c_ expression in 32D cells (murine cells) (top panel, lane 2 vs. lane 1) but not in Mv1Lu cells (mink lung cells) (top panel, lane 4 vs. lane 3). The analysis was performed in triplicates. The data are mean ± SD *Significantly lower than that of cells treated with control siRNA (−): *p *< 0.01

Since OA blocked the activity of the IGFBP‐3‐stimulated PPase and the TGF‐β‐stimulated PPase activity was relatively resistant to inhibition by 0.2–1 nM OA (Figure 1D), OA should be able to reverse IGFBP‐3‐induced growth inhibition, but not TGF‐β‐induce growth inhibition, in Mv1Lu cells. To test this, we determined the effects of OA on growth inhibition (as measured by [Methy‐^3^H] thymidine incorporation) induced by IGFBP‐3 and TGF‐β. As shown in Figure 4A, OA reversed the growth inhibition induced by different concentrations of IGFBP‐3. OA reversed IGFBP‐3‐induced growth inhibition in a concentration‐dependent manner (Figure 4A). At 5 nM, OA reversed IGFBP‐3 (0.1 μg/ml)‐induced growth inhibition by ~80% (Figure 4A) but did not significantly affect TGF‐β‐induced growth inhibition in these cells (Figure 4B). IGFBP‐3‐stimulated PPase is sensitive to OA inhibition, suggesting that IGFBP‐3‐stimulated PPase is likely to be identical to PP2A which is known to be highly sensitive to OA.^26^, ^27^ To test this, we used murine myeloid cells which stably expressed human IR and IRS‐2 (32D cells) and responded to IGFBP‐3‐induced growth inhibition.^22^ PP2A is a heterotrimer composed of a 36‐kDa catalytic C subunit (PP2A_c_), a 65‐kDa scaffolding A subunit, and a 56‐kDa substrate‐recognizing B subunit (PP2A‐B56).^27^ We examined the effect of PP2A**_c_** siRNA transfection on IGFBP‐3‐induced growth inhibition in these murine 32D cells. This PP2A**_c_** siRNA was developed based on the murine sequence. 32D cells were transfected with control siRNA, 2 and 4 nM PP2A**_c_** siRNA by electroporation and treated with IGFBP‐3. As shown in Figure 4C,D, PP2A**_c_** siRNA (2 and 4 nM) reversed the growth inhibition induced by IGFBP‐3 (Figure 4C), but not by TGF‐β (Figure 4D), in a dose‐dependent manner in murine 32D cells. Four nM PP2A**_c_** siRNA reversed IGFBP‐3‐induced growth inhibition at 0.1 μg/ml by ~78% in these murine cells (Figure 4C). This degree of inhibition is comparable to the ~70% downregulation of PP2A**_c_** protein by murine PP2A**_c_** siRNA transfection (vs. control siRNA transfection) (Figure 4E) as determined by 7.5% SDS‐PAGE followed by quantitative Western blot analysis of cell lysates from 32D cells transfected with control siRNA (‐) and 4 nM murine PP2A**_c_** siRNA (+) (Figure 4E, top panel, lane 2 vs. lane 1 and bottom panel, quantitative analysis in three independent experiments). Murine PP2A**_c_** siRNA was unable to reverse IGFBP‐3‐induced growth inhibition in mink Mv1Lu cells (data not shown). This is consistent with the inability of murine PP2A**_c_** siRNA to significantly downregulate mink PP2A**_c_** (Figure 4E, top panel, lane 4 vs. lane 3). These results suggest that IGFBP‐3‐induced growth inhibition is reversed by OA in Mv1Lu cells and by murine PP2A_c_ siRNA in murine 32D cells.

### 3.5 IGFBP‐3 stimulates cytoplasm‐to‐nucleus translocation of PP2A in Mv1Lu cells

**FIGURE 5:**
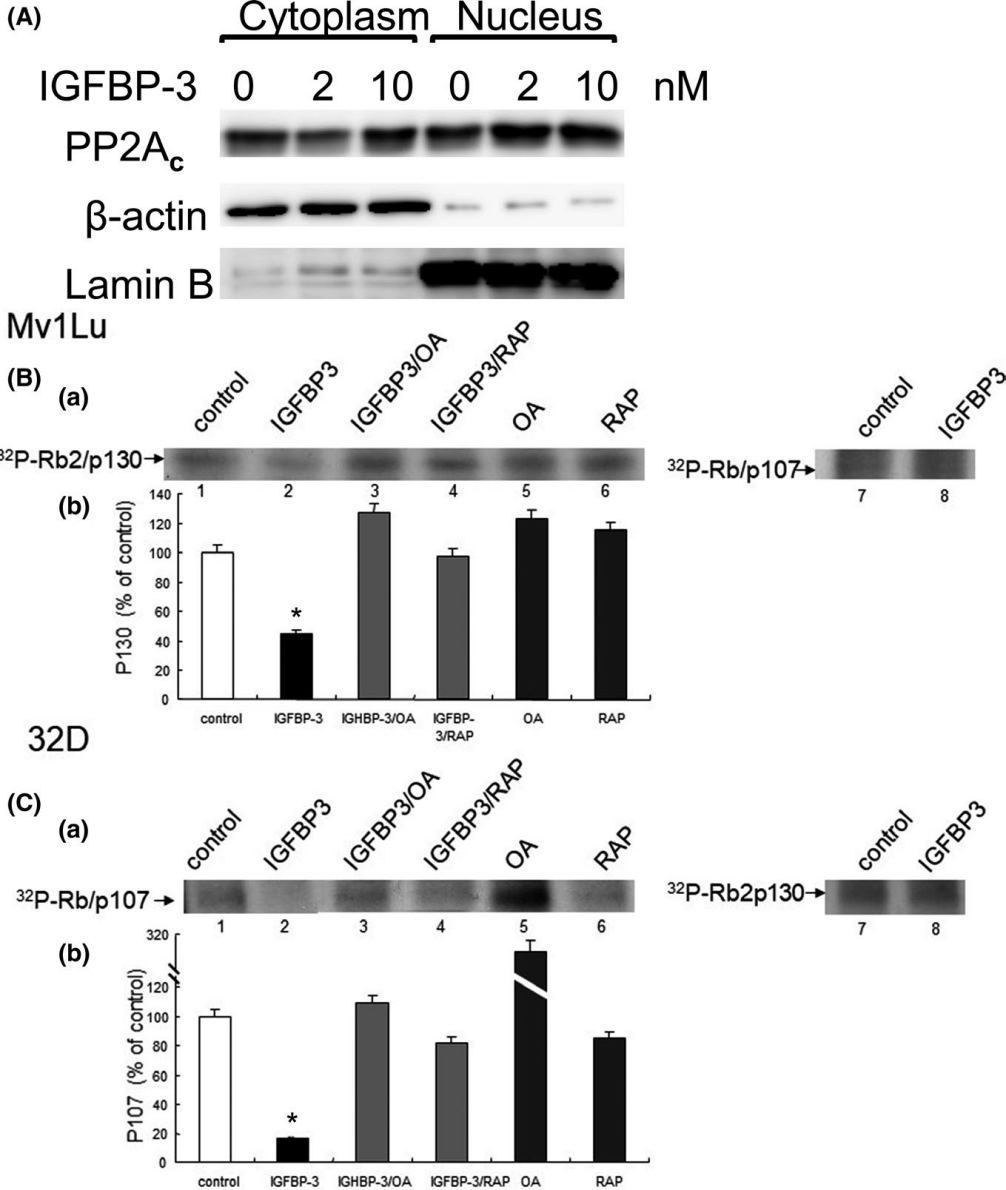
IGFBP‐3 stimulates cytoplasm‐to‐nucleus translocation of PP2A_c_ in Mv1Lu cells (A) and inhibits growth by inducing dephosphorylation of pRb‐related proteins, p130 and p107, in Mv1Lu (B) and 32D (C) cells, respectively. Mv1Lu cells were treated with 0, 2, and 10 nM (or 0, 0.06 and 0.3 µg/ml, respectively), IGFBP‐3 for 2 hr. The cytoplasm and nucleus fractions were separated by centrifugation and analyzed by 7.5% SDS‐PAGE followed by Western blot analysis using antibodies to PP2A_c_, β‐actin, and lamin B. The final volume of the total cytoplasm fraction was 10 times higher than that of the total nucleus fraction. However, an equal volume of cytoplasm and nucleus fractions was analyzed by 7.5% SDS‐PAGE followed by Western blot analysis. At 2 and 10 nM, IGFBP‐3 appeared to increase cytoplasm‐to‐nucleus translocation of PP2A_c_ by ~2 fold. Western blot analysis was the representative of a total of three experiments. Lamin B and β‐actin served as nuclear and cytoplasmic internal standards, respectively. (B and C) Mv1Lu (B) and 32D (C) cells were pre‐incubated with [^32^P]‐orthophosphate for 2 hr, washed and incubated in the culture medium with excess phosphate. ^32^P‐labeled cells were treated with vehicle only or 0.3 µg/ml of IGFBP‐3 in the presence and absence of OA (5 nM) and RAP (60 µg/ml). After 2 hr at 37°C, cell lysates were immunoprecipitated with antibodies to p130 and p107. The immunoprecipitates were analyzed by 7.5% SDS‐PAGE and quantified by a Perkin Elmer phosphorimager (B and C, panels a and b). Phosphorimager analysis was the representative of a total of three experiments. IGFBP‐3 appeared to stimulate dephosphorylation of p130 and p107 in Mv1Lu and 32D cells, respectively (B and C, panels a, lane 2 vs. lane 1 and panel b, quantitative data). OA and RAP inhibited IGFBP‐3‐stimulated dephosphorylation of p130 and p107 (B and C, panel a, lanes 3 and 4 vs. lane 2 and panel b, quantitative data) in Mv1Lu and 32D cells, respectively. The quantitative data from three independent analyses were shown. The data are mean ±SD *Significantly lower than that of cells treated with vehicle only (control): *p *< 0.01

As described above, IGFBP‐3‐stimulated PPase activity and IGFBP‐3‐induced growth inhibition are blocked or reversed by co‐treatment with very low concentrations of OA in Mv1Lu and 32D cells and by transfection with PP2A**_c_** siRNA in murine 32D cells. These suggest that the IGFBP‐3‐stimulated PPase is identical to PP2A. We hypothesized that IGFBP‐3 induces growth inhibition by stimulating IRS‐2‐dependent activation and cytoplasm‐to‐nucleus translocation of PP2A**_c_** in Mv1Lu cells. To test this, Mv1Lu cells were treated with 0, 2, and 10 nM (or 0.06 and 0.3 µg/ml, respectively), IGFBP‐3 for 2 hr. The cytoplasm and nucleus fractions in treated cells were then isolated and subjected to 7.5% SDS‐PAGE followed by Western blot analysis. As shown in Figure 5A, IGFBP‐3 at 2 and 10 nM increased accumulation of PP2A**_c_** in the nucleus fraction by 1.5‐ to 1.7‐fold (n = 3) as compared to that in cells treated with vehicle only (0 nM IGFBP‐3). These results suggest that IGFBP‐3 promotes cytoplasm‐to‐nucleus translocation of PP2A**_c_** (likely as the IRS‐2 complex) in Mv1Lu cells.

### 3.6 IGFBP‐3 inhibits growth by inducing dephosphorylation of pRb‐related proteins, p130 and p107, in Mv1Lu and 32D cells, respectively

PP2A plays a critical multi‐faceted role in the regulation of the cell cycle. It has been implicated in dephosphorylation of two retinoblastoma protein (pRb)‐related proteins, p130 and p107, which interact primarily with E2F4 and E2F5 and are most active in G0‐the quiescent phase of the cell cycle.^26^, ^27^ Moreover, pRb (p105) interacts primarily with E2F1–3 and is most active at the G1‐to‐S phase transition.^26^, ^27^ These suggest that IRS‐2‐PP2A complexes may dephosphorylate pRb‐related proteins (p130 and p107) in the nucleus of target cells. To test this possibility, Mv1Lu and 32D cells were pre‐labeled with ^32^P‐orthophosphate at 37°C for 1 hr, washed, and incubated with 0.3 µg/ml (10 nM) IGFBP‐3 in the presence of excess unlabeled orthophosphate in the medium. After 2 hr at 37°C, ^32^P‐labeled cell lysates were immunoprecipitated with specific antibodies to p130 and p107 and analyzed by 7.5% SDS‐PAGE and quantified by a phosphorimager (panels a and b, respectively). As shown in Figure 5B and Figure 5C, IGFBP‐3‐induced dephosphorylation of p130 and p107 in Mv1Lu and 32D cells, respectively (panel a, lane 2 vs. lane 1 and panel b, quantitative analysis from three experiments). The IGFBP‐3‐induced dephosphorylation of p130 and p107 was blocked in these cells co‐treated with OA and RAP (Figure 5B,C, panel a, lanes 3 and 4 vs. lane 2 and panel b, quantitative analysis from three experiments).

### 3.7 TGF‐β induces colocalization of TβR‐V and PP1_c_ at the plasma membrane and accumulation of PP1 and decreased levels of hyperphosphorylated pRb (P‐Rb) in the nucleus in Mv1Lu cells

**FIGURE 6:**
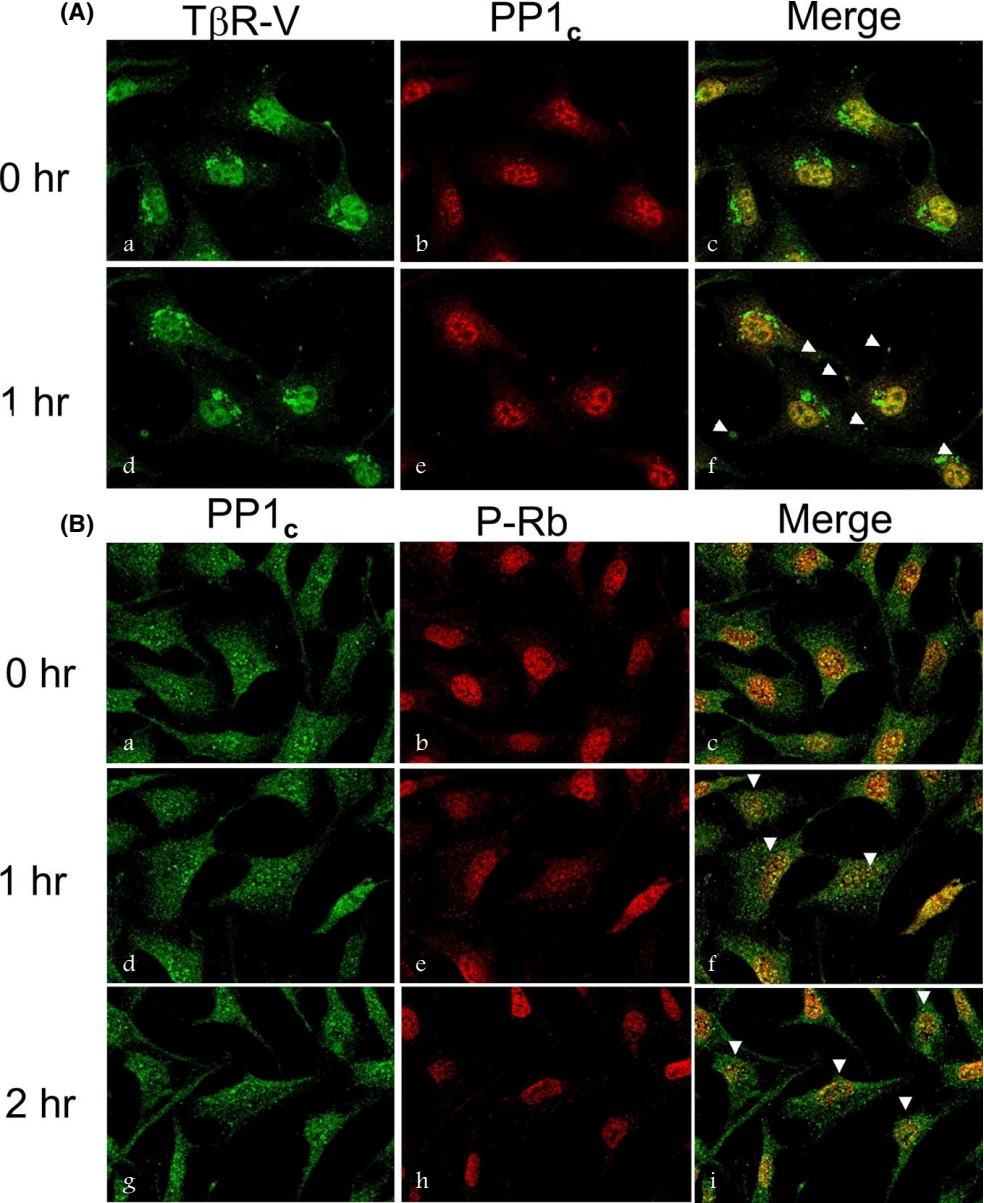
TGF‐β induces co‐localization of TβR‐V and PP1_c_ at the plasma membrane (A) and accumulation of PP1_c_ and decreased levels of hyperphosphorylated pRb (P‐Rb) in the nucleus (B) in Mv1Lu cells. Mv1Lu cells were grown to 50% confluence on coverslips in 35 mm culture dishes at 37˚C for 24 hr. Mv1Lu cells were then treated with 40 pM TGF‐β at 37°C. After 0 and 1 hr (A) or 0, 1, and 2 hr (B), cells were fixed and stained by immunofluorescence using antibodies to TβR‐V and PP1_c_ (A) or using antibodies to PP1_c_ and P‐Rb (B). (A) After immunofluorescence staining, cells on coverslips were counted. Cells treated with TGF‐β at 37°C for 0 hr did not exhibit colocalization of TβR‐V and PP1_c_ at the plasma membrane (Ac). However, cells treated with TGF‐β at 37°C for 1 hr exhibited colocalization of TβR‐V and PP1_c_ at the plasma membrane as indicated by arrowheads (Af). TβR‐V (LRP‐1) is known to undergo constitutive endocytosis and recycling in cells. Perinuclear labeling is likely to be endocytic vesicles which are often seen in juxtanuclear regions. This appearance of endocytic vesicles might be due to longer‐time cell culture before the experiment. (B) After treatment of cells with TGF‐β for 1 or 2 hr at 37°C, approximately 40%–50% cells on a coverslip exhibited significantly decreased yellow fluorescence (co‐localization) in the nucleus, whereas ~90% cells (treated with TGF‐β at 37°C for 0 hr) on a coverslip exhibited yellow fluorescence (colocalization) in the nucleus (Bf,i and Bc, respectively). Arrowheads indicate decreased colocalization (as marked by decreased yellow fluorescence) of PP1_c_ and P‐Rb (Bf,i) due to decreased P‐Rb (as marked by decreased red fluorescence) in the nucleus (Be,h)

The TGF‐β‐stimulated PPase involved in TGF‐β‐induced growth inhibition has been identified as PP1 in human keratinocytes.^28^ PP1 is responsible for dephosphorylating of pRb (p105) which is linked to TGF‐β‐induced growth inhibition in Mv1Lu cells.^29^ The mechanism by which TGF‐β stimulates PP1 activity is not clear. Since TGF‐β induces growth inhibition by stimulating complex formation of TβR‐V, IRS‐1/2, and likely PP1 at the plasma membrane in Mv1Lu cells, we hypothesize that PP1 should be activated by its interaction with IRS‐1/2 in the formation of the TβR‐V‐IRS‐1/2‐PP1 ternary complexes in TGF‐β‐treated cells. PP1 enzyme contains both a 37‐kDa catalytic subunit (PP1_c_) and at least one regulatory subunit which directs PP1_c_ to different substrates or sites. To test this, we performed immunofluorescence microscopy in Mv1Lu cells treated with 40 pM TGF‐β at 37˚C for 0 and 1 hr using antibodies to TβR‐V (LRP‐1) and PP1_c_ (Figure 6A). TGF‐β‐stimulated colocalization of TβR‐V and PP1_c_ at the plasma membrane, as indicated by arrowheads in Mv1Lu cells treated with TGF‐β at 37˚C for 1 hr (Figure 6Af). In contrast, Mv1Lu cells treated with 40 pM TGF‐β for 0 hr did not exhibit colocalization of TβR‐V and PP1_c_ in these cells (Figure 6Ac).

Retinoblastoma protein (pRb) present in the cytoplasm and nucleus fractions are identified as hyperphosphorylated (as a slow‐migrating form) and hypophosphorylated (as a fast‐migrating form) forms of pRb, respectively, based on its mobility on 7.5% SDS‐PAGE.^29^, ^30^, ^31^ Cytoplasmic pRb is known to be mainly the hyperphosphorylated form.^32^ These suggest that TGF‐β stimulates cytoplasm‐to‐nucleus translocation of PP1_c_ and correspondingly increases the amount of hypophosphorylated pRb (as a fast‐migrating form of pRb on 7.5% SDS‐PAGE), which is the PP1_c_‐dephosphorylated product of pRb in the nucleus. To demonstrate the subsequent cytoplasm‐to‐nucleus translocation of PP1_c_ and its effect on dephosphorylation of pRb in the nucleus, we performed immunofluorescence analysis in Mv1Lu cells treated with 40 pM TGF‐β at 37˚C for 0, 1, and 2 hr using specific antibodies to PP1_c_ and hyperphosphorylated pRb (P‐Rb). We reasoned that TGF‐β promotes nucleus accumulation of PP1_c_ and should accordingly decrease the amount of P‐Rb, its target substrate, in the nucleus. PP1 specifically dephosphorylates pRb in the nucleus of target cells.^28^, ^29^ After treatment of cells with TGF‐β and immunofluorescence staining, six images, which consist of 6–8 cells/image, were taken at different areas of cells grown on a coverslip. The image shown in the data was the representative of the six images. As shown in Figure 6B, after treatment of cells with TGF‐β at 37˚C for 1 or 2 hr, approximately 40%–50% cells on a coverslip exhibited significantly decreased yellow fluorescence (co‐localization) in the nucleus, whereas ~90% cells (treated with TGF‐β at 37˚C for 0 hr) on a coverslip exhibited yellow fluorescence (colocalization) in the nucleus (Figure 6Bf,i and Figure 6Bc, respectively). TGF‐β treatment of cells for 1 and 2 hr decreased the amount of P‐Rb and colocalization of PP1_c_ and P‐Rb in the nucleus (Figure 6Be,h and Figure 6Bf,i, respectively). These results support the suggestion that TGF‐β promotes cytoplasm‐to‐nucleus translocation of PP1_c_, resulting in dephosphorylation of pRb in the nucleus, which leads to cell growth arrest.

### 3.8 TGF‐β stimulates cytoplasm‐to‐nucleus translocation of PP1_c_ and increases formation of dephosphorylated pRb (Rb) in the nucleus in Mv1Lu (A) and A549 (B) cells

**FIGURE 7:**
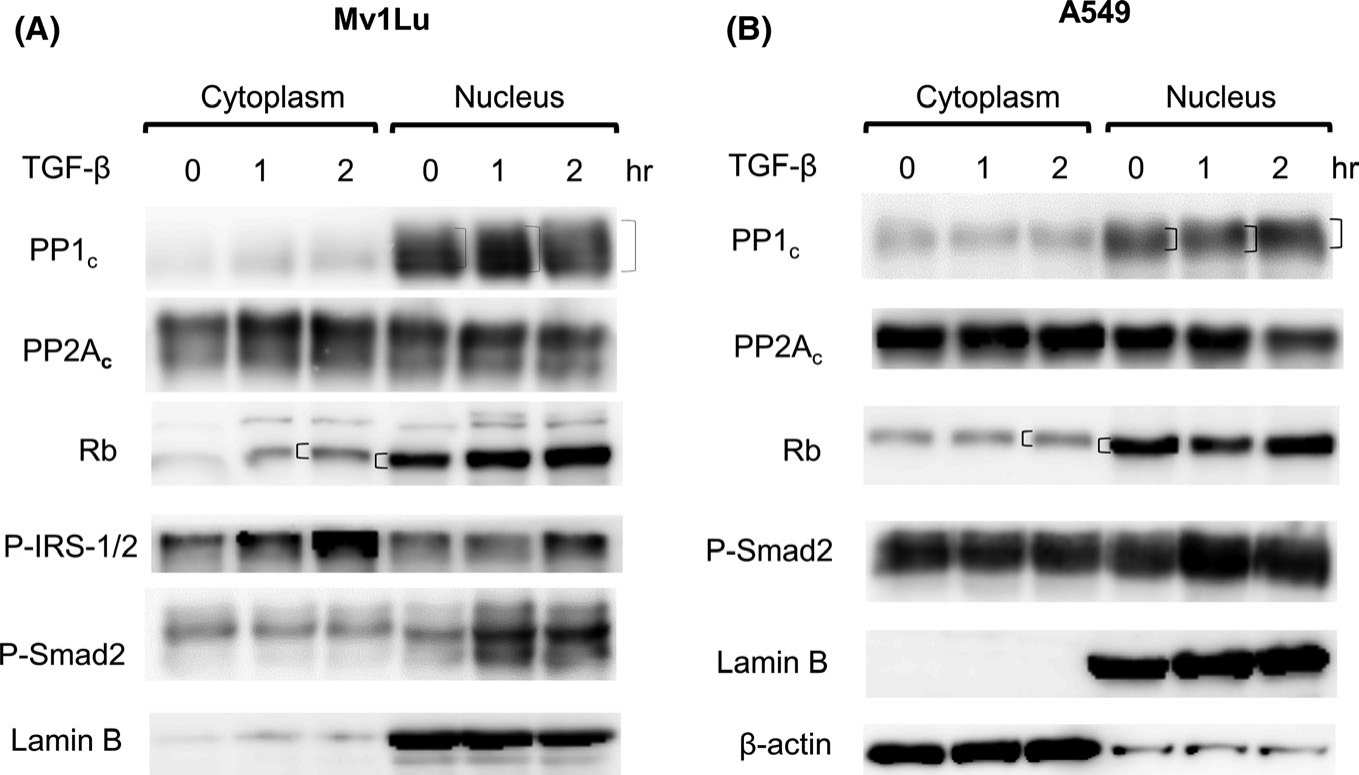
TGF‐β stimulates cytoplasm‐to‐nucleus translocation of PP1_c_ in Mv1Lu (A) and A549 (B) cells and increases dephosphorylated pRb (a fast‐migrating form of pRb) levels in the nucleus in these cells. Mv1Lu (A) and A549 (B) cells were treated with 40 pM TGF‐β for 0, 1, and 2 hr. The cytoplasm and nucleus fractions were separated by centrifugation and analyzed by 7.5% SDS‐PAGE followed by quantitative Western blot analysis using antibodies to PP1_c_, PP2A_c_, pRb (Rb), P‐IRS‐1/2 (phosphorylated IRS‐1/2, Ser 270), P‐Smad2 (phosphorylated Smad2), lamin B, and β‐actin. pRb (Rb) (present in the nucleus) migrated as a fast‐migrating form (dephosphorylated pRb) on 7.5% SDS‐PAGE. pRb (Rb) (present in the cytoplasm) migrated as a slow form (phosphorylated pRb). The final volume of the total cytoplasm fraction was 10 times higher than that of the total nucleus fraction. An equal volume of cytoplasm and nucleus fractions was then analyzed by 7.5% SDS‐PAGE followed by Western blot analysis. Western blots were representatives of a total of three experiments. Lamin B and β‐actin served as nuclear and cytoplasmic internal standards, respectively

To further support the hypothesis that TGF‐β stimulates cytoplasm‐to‐nucleus translocation of PP1, we determined the subcellular localization of PP1_c_, PP2A_c_, pRb (Rb), phosphorylated Smad2 (P‐Smad2), phosphorylated IRS‐1/2 (P‐IRS‐1/2, phosphorylation at Ser 270), lamin B, and β‐actin using 7.5% SDS‐PAGE and quantitative Western blot analysis with specific antibodies to PP1_c_, PP2A_c_, and others after subcellular cytoplasm/nucleus fractionation of Mv1Lu and A549 cells treated with 40 pM TGF‐β for 0, 1, and 2 hr. As shown in Figure 7, TGF‐β increased the amounts of PP1_c_ (Figure 7A,B), dephosphorylated Rb (as a fast‐migrating form of Rb on 7.5% SDS‐PAGE) (Figure 7A,B), P‐IRS‐2 (Figure 7A), and P‐Smad2 (Figure 7A,B) in the nucleus fraction in a time‐dependent manner in these cells. Both P‐IRS‐1/2 contain Ser 270 but only P‐IRS‐2 entered the nucleus (Figure 3Be,h). After 2 hr, TGF‐β increased the amounts of PP1_c_, dephosphorylated Rb (as a fast migrating form on 7.5% SDS‐PAGE), P‐IRS‐2 and P‐Smad2 by 1.5‐ to 2‐fold (n = 3) in the nucleus fraction in Mv1Lu and A549 cells. In contrast, TGF‐β did not significantly increase the amount of PP2A_c_ in the nucleus fraction in these cells. Interestingly, Rb present in the cytoplasm and nucleus fractions were identified as phosphorylated (as a slow‐migrating form) and dephosphorylated (as a fast‐migrating form) forms of Rb, respectively, based on its mobility on 7.5% SDS‐PAGE.^29^, ^30^, ^31^ Cytoplasmic Rb is known to be mainly the hyperphosphorylated form.^32^ These results suggest that TGF‐β stimulates cytoplasm‐to‐nucleus translocation of PP1_c_ and P‐IRS‐2, and correspondingly increases the amount of dephosphorylated Rb (as a fast‐migrating form of Rb), which is the PP1‐dephosphorylated product of Rb, in the nucleus.

## 4 DISCUSSION

**FIGURE 8:**
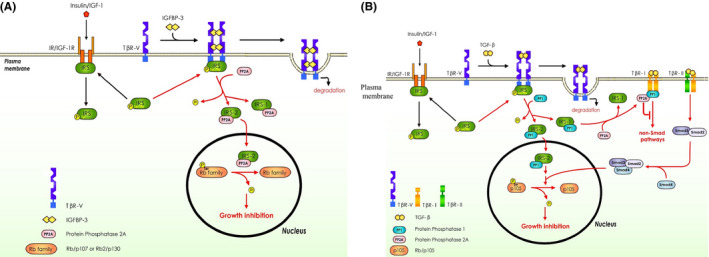
Models for the mechanisms by which IGFBP‐3 (A) and TGF‐β (B) induces cellular growth inhibition and the cross talk between TβR‐V and IR/IGF‐1R signaling (A, B). (A)​ IGFBP‐3 induces growth inhibition by stimulating TβR‐V‐mediated IRS‐2‐dependent activation and cytoplasm‐to‐nucleus translocation of PP2A, resulting in dephosphorylation of pRb‐related proteins (p130 and p107). In the IGFBP‐3‐stimulated tumor suppressor signaling cascade (TβR‐V/IRS‐2/PP2A/p107, p130), TβR‐V is identical to LRP‐1 which has a 515‐kDa α chain that contains four cell surface ligand‐binding subdomains (I, II, III, and IV) and an 85‐kDa β chain comprising the transmembrane domain and cytoplasmic tail. IGFBP‐3 binds to cell surface subdomains II and IV of TβR‐V. Insulin and IGF‐I antagonize IGFBP‐3‐induced cellular growth inhibition by stimulating tyrosine phosphorylation of IRS‐1/2 via interaction with their cognate receptors. The signaling cascades potentiated in diabetic patients are indicated by red arrows. Under non‐insulin‐stimulating conditions, IRS‐1/2 are mainly phosphorylated at serine residues. (B) In epithelial cells, TGF‐β induces potent growth inhibition (100% growth inhibition at 1–5 pM) by stimulating the TβR‐V‐mediated IRS‐2‐dependent tumor suppressor signaling cascade (TβR‐V/IRS‐2/PP1/pRb) in concert with canonical TGF‐β signaling mediated by TβR‐I and TβR‐II (TβR‐I/TβR‐II/Smad‐2/3/4). The TβR‐V‐mediated tumor suppressor signaling cascade (TβR‐V/IRS‐2/PP1/pRb) is essential to dephosphorylate (activate) pRb (by PP1) for causing TGF‐β growth inhibition. TβR‐I–TβR‐II‐mediated canonical signaling is required for potentiating TGF‐β growth inhibition (mediated by TβR‐V) by transcriptional activation of CDK inhibitors^14^ which activate the TβR‐V/IRS‐2/PP1/pRb cascade via maintaining pRb unphosphorylated (active) in the nucleus.^7^ TGF‐β also stimulates IRS‐1‐dependent activation and formation of PP1‐PP2A complexes which target and suppress TβR‐I‐mediated non‐Smad pathways. The TβR‐I is present as TβR‐V–TβR‐I complexes^9^ mainly localized in plasma‐membrane non‐lipid raft microdomains. In normal epithelial cells, which express TβR‐V, TGF‐β, as a tumor suppressor, suppresses carcinogenesis by potently inhibiting cell growth in normal epithelial cells via stimulating TβR‐V‐mediated IRS‐2‐dependent tumor suppressor signaling (TβR‐V/IRS‐2/PP1/pRb) in concert with canonical TGF‐β signaling (TβR‐I/TβR‐II/Smad‐2/3/4) and by suppressing TβR‐I‐mediated tumor progression via stimulating TβR‐V‐mediated IRS‐1‐dependent signaling (TβR‐V/IRS‐1/PP1‐PP2A/TβR‐I). Insulin and IGF‐I antagonize TGF‐β‐stimulated TβR‐V/IRS‐1/2/PP1 signaling by stimulating tyrosine phosphorylation of IRS‐1/2 via interaction with their cognate receptors, insulin receptor (IR), and IGF‐1 receptor (IGF‐1R). The tyrosine phosphorylation of IRS‐1/2 catalyzed by IR and IGF‐1 receptor, which occurs more rapidly than the Ser/Thr‐specific dephosphorylation of IRS‐1/2 by PP1 or PP2A, leads to multiple IR/IGF‐1R downstream signaling pathways and prevents the formation of TβR‐V‐IRS‐1/2‐PP1 complexes. Tyrosine phosphorylation and Ser/Thr‐specific dephosphorylation of IRS‐1/2 are mutually exclusive.^7^ In diabetes, insulin or insulin signaling defects potentiate TGF‐β‐induced growth inhibition in target cells.^7^ In addition, high glucose in the plasma and tissues of diabetic patients may enhance TβR‐V and TβR‐I/TβR‐II signaling via increasing TGF‐β production and TβR‐I/TβR‐II expression.^7^, ^80^ Increased ECM synthesis (which is mediated by TβR‐I/TβR‐II/Smad2/3/4 signaling) further attenuates insulin signaling and enhances TGF‐β‐induced growth inhibition,^7^ resulting in alopecia, impaired wound healing, accelerated glomerulopathy, and tissue fibrosis in diabetic patients. The signaling cascades potentiated in diabetic patients are indicated by red arrows

Here, we have provided evidence revealing that IGFBP‐3 inhibits growth in epithelial cells by stimulating the TβR‐V‐mediated growth inhibition signaling cascade which involves IRS‐2, PP2A, and pRb‐related proteins, p130 or p107. We propose a revised version of our previously published model^7^ to demonstrate the molecular mechanism of IGFBP‐3‐induced growth inhibition in epithelial cells and other cell types. In this model (Figure 8A), IGFBP‐3 is a non‐covalently linked homodimeric protein. It binds to TβR‐V by interaction with its cell surface subdomains II and IV,^4^, ^5^, ^7^ resulting in dimerization of TβR‐V and subsequent recruitment of Ser/Thr‐phosphorylated IRS‐1 or IRS‐2 and then PP2A to the cytoplasmic tail of the dimeric TβR‐V. Interacting proteins or specific protein substrates of PP2A are known to possess PP2A‐B56 docking (or binding) motifs (L/MxxL/I/VxE) which contain contiguous six amino acid residues and are well conserved throughout the eukaryotic domain of life and in human viruses.^33^ IRS‐1 and IRS‐2 possess PP2A‐B56 docking motifs of LytrdE (residues 86–91) and LkeLfE (residues 294–299), respectively, which provide binding sites for PP2A‐B56 (binding affinity: IRS‐2 > IRS‐1). PP2A in the TβR‐V‐IRS‐1‐PP2A or TβR‐V‐IRS‐2‐PP2A ternary complex becomes activated and dephosphorylates IRS‐1 or IRS‐2 in the complex, leading to dissociation of dephosphorylated IRS‐1‐PP2A or IRS‐2‐PP2A binary complexes from the cytoplasmic tail of TβR‐V (at the plasma membrane). High‐affinity‐bound dephosphorylated IRS‐2‐PP2A complexes then undergo IRS‐2‐dependent translocation from cytoplasm to the nucleus where activated PP2A dephosphorylates pRb‐related proteins, p130 and p107, resulting in growth arrest. p130 and p107 possess high‐affinity PP2A‐B56 docking motifs of LsgIlE (residues 519–524) and LinIfE (residues 412–417), respectively. It is important to note that p130 and p107 do not possess specific PP1_c_ docking motifs (FxxR/KxR/K),^34^ suggesting that p130 and p107 are the PP2A target substrates in the nucleus. In this communication, we also demonstrate that IGFBP‐3 stimulates cytoplasm‐to‐nucleus translocation of IRS‐2 but not IRS‐1. IRS‐2 has been shown to undergo nuclear translocation in normal and cancer cells.^35^ It possesses a putative nuclear localization signal (NLS) motif of KKwRsK (residues 80–85). Moreover, after dissociation from the cytoplasmic tail of TβR‐V, low‐affinity‐bound dephosphorylated IRS‐1‐PP2A complexes are mainly present in the cytoplasm and do not have known functions in cells treated with IGFBP‐3.^7^ However, IGFBP‐3 is known to inhibit phosphorylation of c‐raf‐MEK‐ERK and p38 kinase in insulin‐secreting cells.^36^ PP2A is also known to inhibit the kinase activities of the kinases involved in TβR‐I‐activated non‐Smad pathways, which include JNK,^37^ TAK1‐p38/JNK,^38^ PI3K‐AKT,^39^ and RhoA‐Rock^40^ signaling, by dephosphorylating these kinases.^41^, ^42^ It is likely that IRS‐1‐PP2A is responsible, at least in part, for IGFBP‐3‐induced inhibition of non‐Smad signaling.^36^

Several lines of evidence suggest that IGFBP‐3 acts as a potential tumor suppressor gene. First, aberrant promoter methylation of IGFBP‐3 gene, which silences its expression, is detected in human gastric cancer, colorectal cancer, breast cancer, and malignant mesothelioma cancer.^20^ Second, low levels of IGFBP‐3 expression in cancer tissues are correlated with poor prognosis for patients with esophageal squamous cell carcinoma^43^ and hepatocellular carcinoma.^44^ Third, low IGFBP‐3 expression correlates clinically with higher tumor grade, advanced stage, and poor survival in ovarian endometrioid adenocarcinoma patients.^45^ Here, we demonstrate that IGFBP‐3 inhibits cell growth by stimulating the TβR‐V‐mediated tumor suppressor signaling pathway (TβR‐V/IRS‐1/2/PP2A/p130, p107). Among the components of this signaling cascade, TβR‐V and PP2A have been proved to be tumor suppressor genes by that stable transfection of human carcinoma cells and CHO‐LRP‐1^−/−^ cells with LRP‐1 (TβR‐V) cDNA restores the growth inhibitory response to IGFBP‐3 and TGF‐β, and normal epithelial morphology^10^, ^15^ and by that loss of PP2A regulatory subunit B56δ promotes spontaneous tumorigenesis in vivo.^46^ In addition, knockout of the TβR‐V (LRP‐1) gene and both p130 and p107 genes in mice has been shown to cause embryonic and neonatal lethality, respectively.^47^, ^48^ In normal epithelial cells, IGFBP‐3 induces growth inhibition by stimulating TβR‐V‐mediated and IRS‐1/2‐dependent activation of PP2A. PP2A may serve as an important down‐stream effector for mediating other known cell biological activities of IGFBP‐3 and other high‐affinity IGFBPs.^1^, ^36^

TβR‐V is the only cell surface IGFBP‐3 receptor identified by I^125^‐labeled IGFBP‐3 affinity labeling (binding/cross‐linking) followed by immunoprecipitation using antiserum to TβR‐V in epithelial cells and other cell types.^4^, ^5^, ^6^ TβR‐V also binds IGFBP‐4 and IGFBP‐5 but not IGFBP‐1, IGFBP‐2, and IGFBP‐6, as determined by I^125^‐labeled IGFBPs affinity labeling.^5^, ^6^ It exhibits the highest binding affinity toward IGFBP‐3 with a K_d_ of 10 nM.^4^, ^5^, ^6^ A TGF‐β peptide antagonist β_1_^25^, which contains a minimal active site motif of WS/CXD in TGF‐β and IGFBP‐3 molecules, blocks TGF‐β and IGFBP‐3 binding to TGF‐β receptors in epithelial cells and reverses growth inhibition induced by either TGF‐β or IGFBP‐3 in these cells.^4^, ^5^, ^11^, ^12^ The transmembrane protein TMEM219 (25 kDa) was also identified as an IGFBP‐3 receptor (termed IGFBP‐3R) in 2010, using the yeast two‐hybrid screening and a human breast cancer cell cDNA library.^49^ In contrast to the IGFBP‐3 receptor (TβR‐V/LRP‐1), IGFBP‐3R/TMEM219 does not bind other high‐affinity IGFBPs^49^ and have known function of a tumor suppressor gene. IGFBP‐3R/TMEM219 was identified as a cell death receptor mediating IGFBP‐3‐induced anti‐tumor effects in cancer cells^49^, ^50^ and as an autophagy‐activation receptor mediating IGFBP‐3‐activated autophagy in Vero cells.^51^ In addition, the K_d_ of IGFBP‐3 binding to TMEM219/IGFBP‐3R has been estimated to be 125 nM.^51^ IGFBP‐3 (1 µM) is utilized to stimulate TMEM219/IGFBP‐3R‐mediated autophagy activation in Vero cells (kidney epithelial cells).^51^ It is important to note that carcinoma cancer cells do not express the IGFBP‐3 receptor (TβR‐V) and loss of TβR‐V confers cancer malignancy.^7^ Many lines of evidence suggest that the IGFBP‐3 receptor (TβR‐V) is the primary IGFBP‐3 receptor in normal epithelial cells and other cell types.^7^

The transcriptional activation and growth inhibition activities of TGF‐β have generally been thought to be mediated by the canonical TβR‐I/TβR‐II/Smad2/3/4 signaling cascade.^13^ However, these two activities appear to segregate in several cell types and under various conditions.^52^, ^53^, ^54^ This suggests that other signaling pathways must be involved in mediating the TGF‐β activities. Although TGF‐β‐stimulated canonical TβR‐I/TβR‐II/Smad2/3/4 signaling can be modulated by other signaling pathways,^55^, ^56^ it is mainly responsible for mediating the transcriptional activation of ECM synthesis‐related genes. Smad2/3/4 responsive elements exist in the promoter regions of all responsive genes. In contrast, the signaling involved in TGF‐β‐induced growth inhibition in target cells is unknown. Other signaling pathways, in addition to the well‐known canonical TβR‐I/TβR‐II/Smad2/3/4 signaling pathway, are suggested to be involved in the growth inhibitory response to TGF‐β.^57^, ^58^ The Ras/ERK signaling and PP2A are involved in mediating TGF‐β‐induced growth inhibition in certain cells.^59^, ^60^ However, the main signaling pathway, in concert with canonical TβR‐I/TβR‐II/Smad2/3/4 signaling,^13^ mediates the growth inhibitory response to TGF‐β in epithelial cells remains unknown. TβR‐I, TβR‐II, TβR‐III, and TβR‐V co‐express in all normal cell types studied. Since the TβR‐III null mutation in mice does not affect the growth regulatory response to TGF‐β in embryonic fibroblasts derived from these mice,^61^ the remaining candidate is TβR‐V. Many carcinoma cells and primary tumors express no or very low levels of TβR‐V expression.^2^, ^7^, ^16^, ^17^, ^18^ Growth of these cells is not inhibited by either TGF‐β or IGFBP‐3. In the absence of TβR‐V in late‐stage cancer, TGF‐β induces EMT (epithelial–mesenchymal transition), autoinduction, and increased invasiveness by stimulating TβR‐I‐activated or TβR‐I‐mediated non‐Smad signaling pathways^37^, ^38^, ^39^, ^40^ as well as canonical Smad signaling (TβR‐I/TβR‐II/Smad2/3/4 signaling).^13^ TβR‐II is apparently not involved in TGF‐β‐stimulated TβR‐I‐activated/mediated non‐Smad signaling pathways^37^, ^38^, ^39^, ^40^, ^41^, ^42^ which are involved in cell survival, migration, proliferation, malignant transformation, and tumor growth. In fact, TGF‐β stimulates tumor promoter signaling toward EMT is mediated by both non‐Smad and Smad pathways. While no LRP‐1 (TβR‐V) is detected in hepatoma in human patients, it is present in the normal parenchymal tissue surrounding the hepatomas.^16^ These suggest that TβR‐V is involved in mediating the growth inhibitory response to TGF‐β in normal epithelial cells and that its loss contributes to the malignant phenotype in cancer cells.^7^ These are also consistent with the notion that TβR‐V acts as a tumor suppressor gene and controls cell growth in normal epithelial cells by mediating TGF‐β‐induced growth inhibition in these cells. The loss or deficiency of TβR‐V leads to the development of carcinoma cancer. Although no mutation in the LRP‐1 (TβR‐V) gene has been found related to cancer initiation or progression, the T allele of the C766 T polymorphism in the LRP‐1 (TβR‐V) gene is associated with an increased risk of breast cancer.^62^

We previously proposed a model for the mechanism by which TGF‐β inhibits growth in epithelial cells by binding to a site between cell surface subdomains I and II of TβR‐V in target cells. In this model, TGF‐β stimulates sequential association of IRS‐1 or IRS‐2 and a Ser/Thr‐specific PPase with the cytoplasmic tail of TβR‐V by inducing TβR‐V dimerization via its covalently linked homodimeric structure. In the ternary complexes, the Ser/Thr‐specific PPase becomes activated and dephosphorylate IRS‐1/2. Dephosphorylated IRS‐1‐PPase or IRS‐2‐PPase binary complexes dissociate from the cytoplasmic tail of TβR‐V and undergo IRS‐1/2‐dependent translocation from cytoplasm to the nucleus where the PPase dephosphorylates pRb (retinoblastoma protein) or pRb‐related proteins, resulting in growth arrest. This model lacked the identity of PPase, IRS‐1/2, and retinoblastoma family proteins which are involved in TGF‐β‐stimulated TβR‐V‐mediated tumor suppressor (growth inhibition) signaling.^7^ Here, we provide several lines of evidence to suggest that PP1, IRS‐1/2, and pRb (p105) are involved in the TGF‐β‐stimulated TβR‐V‐mediated tumor suppressor signaling cascade (TβR‐V/IRS‐2/PP1/pRb). These include: (1) TGF‐β stimulates colocalization of TβR‐V and IRS‐1/2 at the plasma membrane in Mv1Lu cells, as demonstrated by immunofluorescence staining. (2) TGF‐β stimulates cytoplasm‐to‐nucleus translocation of IRS‐2 but not IRS‐1 in Mv1Lu cells, as demonstrated by immunofluorescence staining. (3) PP1 is known to be responsible for mediating TGF‐β‐stimulated dephosphorylation of pRb in keratinocytes and Mv1Lu cells.^28^, ^29^ (4) TGF‐β‐stimulated PPase (PP1) activity is abolished in cells co‐treated with RAP (LRP‐1/TβR‐V antagonist) or insulin^7^, ^22^, ^23^ in Mv1Lu cells. (5) TGF‐β‐stimulated PPase (PP1) activity is distinct from IGFBP‐3‐stimulated PPase (PP2A) activity in its relative insensitivity to OA inhibition, which appears to be the biochemical character of PP1 activity.^26^, ^27^ PP2A is completely inhibited at 1 nM OA, compared to greater than 1 µM OA for PP1.^26^ (6) OA at 0.5 nM completely inhibits IGFBP‐3‐stimulated PPase (PP2A) activity, but not TGF‐β‐stimulated PPase (PP1) activity, in Mv1Lu cells treated with IGFBP‐3 and TGF‐β. (7) TGF‐β stimulates cytoplasm‐to‐nucleus translocation of PP1_c_, resulting in dephosphorylation of pRb (p105) in the nucleus, as demonstrated by Western blot analysis following subcellular fractionation (to yield cytoplasm and nucleus fractions) and immunofluorescence analysis. (8) PP1 as well as PP2A are the master regulators of the eukaryotic cell cycle.^27^ The above evidence supports an updated model (Figure 8B) in which TGF‐β induces growth inhibition in target cells by stimulating TβR‐V‐mediated signaling (TβR‐V/IRS‐2/PP1) which leads to dephosphorylation of pRb (p105) in the nucleus, resulting in cell growth arrest.

In this model (Figure 8B), TGF‐β, a covalently associated homodimeric cytokine, interacts with TβR‐V at a site between subdomains I and II,^7^, ^10^, ^13^ resulting in dimerization of TβR‐V and sequential recruitment of IRS‐1 or IRS‐2 and PP1 to the cytoplasmic tail of dimeric TβR‐V to form TβR‐V‐IRS‐1‐PP1 or TβR‐V‐IRS‐2‐PP1 ternary complexes. IRS‐1 and IRS‐2 possess PP1_c_ docking motifs of FrssfR (residues 438–443) and FefRpR (residues 298–303), respectively (PP1_c_ docking affinity: IRS‐2 > IRS‐1). IRS‐2 appears to comprise of overlapping high‐affinity PP1 and PP2A docking motifs of FefRpR (residues 298–303) and LkeLfE (residues 294–299), respectively, suggesting that PP1 and PP2A docking to IRS‐2 are mutually exclusive. After dephosphorylation of IRS‐2 by activated PP1 in the ternary complex, dephosphorylated IRS‐2‐PP1 binary complexes dissociate from the cytoplasmic tail of TβR‐V and enter the nucleus via the nucleus‐targeting function of IRS‐2. In the nucleus, PP1 dephosphorylates pRb (p105), resulting in cell growth arrest. In normal epithelial cells, TGF‐β potently inhibits cell growth (~100% growth inhibition at 1–5 pM) by stimulating the TβR‐V‐mediated signaling cascade (TβR‐V/IRS‐2/PP1/pRb) to dephosphorylate (activate) pRb by PP1 in concert with canonical TGF‐β signaling (TβR‐I/TβR‐II/Smad2/3/4).^7^ TGF‐β‐stimulated canonical signaling potentiates TβR‐V‐mediated growth inhibition in these epithelial cells, at least in part, by transcriptional activation of cyclin‐dependent kinase (CDK) inhibitors which maintain pRb unphosphorylated (active) in the nucleus.^7^, ^13^ Moreover, upon TGF‐β stimulation in cells, dephosphorylated IRS‐1‐PP1 complexes also dissociate from TβR‐V and bind (or anchor) to a high‐affinity PP1 docking motif FesfKR (residues 393–398) in the cytoplasmic domain of TβR‐I in the TβR‐V–TβR‐I complex.^9^ Since PP1_c_ itself possesses a high‐affinity PP2A‐B56 docking motif of LlrLfE (residues 82–87), on the way to bind to TβR‐I (Figure 8B), PP1_c_ also recruits PP2A to form the high‐affinity‐bound TβR‐1‐PP_c_‐PP2A complex. PP2A recruited by TβR‐1 as the high‐affinity‐bound PP1‐PP2A complex^63^, ^64^ effectively suppresses or silences TGF‐β‐stimulated TβR‐I‐mediated non‐Smad pathways by dephosphorylation of the kinases involved in non‐Smad pathways in normal epithelial cells. However, in cancer cells lacking TβR‐V expression,^7^ TβR‐I is transiently localized in lipid rafts due to its specific interaction with caveolin‐1, a structural component of lipid rafts.^65^ Lipid rafts serve as major platforms for non‐Smad signaling regulation in cell migration and proliferation.^66^ In these cancer cells, TβR‐I‐mediated non‐Smad signaling pathways are activated by TGF‐β due to defective recruitment of PP‐1_c_‐PP2A by TβR‐I to suppress non‐Smad signaling.^42^ Furthermore, in carcinoma cells, loss or very low levels of TβR‐V expression do not affect TGF‐β‐stimulated canonical signaling (TβR‐I/TβR‐II/Smad‐2/3/4), as evidenced by TGF‐β‐stimulated expression of PAI‐1 in these cells.^10^ Thus, as a tumor promoter, TGF‐β stimulates both Smad and non‐Smad signaling (termed tumor promoter signaling), leading to epithelial mesenchymal transition (EMT), autoinduction, invasiveness, and chemoresistance in these cancer cells. PP2A plays a pivotal role in suppressing the development of cancer malignancy via TGF‐β‐induced TβR‐I recruitment of PP1‐PP2A complexes^41^, ^42^ to suppress non‐Smad signaling pathways.^37^, ^38^, ^39^, ^40^ The expression and activity of PP2A are commonly reduced in cancer tissues. Small molecule PP2A activators have been developed to treat cancer.^67^

TGF‐β is known to act as a tumor suppressor and a tumor promoter during tumorigenesis. The mechanism of switching TGF‐β from a tumor suppressor to a tumor promoter in the process of tumorigenesis remains unclear.^68^ We hypothesize that the presence and absence of TβR‐V expression in target cells appear to be critical in determining whether TGF‐β is a tumor suppressor or a tumor promoter.^7^, ^69^ In normal epithelial cells which express TβR‐V, TGF‐β suppresses carcinogenesis by potently inhibiting cell growth via stimulating TβR‐V‐mediated IRS‐2‐dependent tumor suppressor signaling cascade (TβR‐V/IRS‐2/PP1/pRb) in concert with TβR‐I–TβR‐II‐mediated canonical TGF‐β signaling (TβR‐I/TβR‐II/Smad2/3/4) and by suppressing tumor progression via stimulating TβR‐V‐mediated IRS‐1‐dependent activation and formation of PP1‐PP2A complexes (TβR‐V/IRS‐1/PP1‐PP2A) which targets and suppresses TβR‐I‐mediated non‐Smad tumor progression signaling (Figure 8B). In cancer cells, loss or very low levels of TβR‐V expression cause the inability of TGF‐β to inhibit cell growth but promote tumor growth. In the absence of TβR‐V and PP1‐PP2A complexes in cancer cells, TβR‐I becomes active as a homodimeric protein. In these cells, TGF‐β is able to stimulate TβR‐I‐mediated non‐Smad signaling pathways. Together with canonical Smad signaling,^13^ non‐Smad signaling contributes to TGF‐β‐induced EMT, autoinduction, invasiveness, chemoresistance, and immunomodulation in late‐stage cancer. This raises an interesting question: how much minimum expression threshold of TβR‐V in epithelial cells is required for switching TGF‐β from a tumor suppressor to a tumor promoter. To test this, we used pseudomonas exotoxin treatment of Mv1Lu cells to select mutant cells with reduced expression of TβR‐V (LRP‐1).^10^ We found that PEA‐C11 cells, a representative clone, possess ~15% as much cell surface TβR‐V as parent cells and that TGF‐β, at 40 pM, inhibits cell growth in PEA‐C11 cells by 70% as compared with ~100% inhibition in wild‐type Mv1Lu cells.^10^ These results suggest that relatively low levels of TβR‐V at the cell surface are enough to maintain the status of TGF‐β as a growth inhibitor or TβR‐V as a tumor suppressor in target cells. They also suggest that TβR‐V (LRP‐1)‐knockdown approach (e.g., siRNA) may not be perfect for examining the role of TβR‐V in the cellular function of interest because of possibly enough presence of remaining TβR‐V (~15%) in LRP‐1 knockdown cells.^70^

Activation of tumor suppressors or their signaling for the treatment of human cancers has been a long sought, yet elusive, strategy for an effective therapy.^71^ TβR‐V is the only known membrane receptor which acts as a tumor suppressor required for epithelial cells.^7^, ^69^ More than 80% of human cancers are carcinomas. The TβR‐V‐mediated tumor suppressor signaling cascade should be an ideal target for developing a strategy to prevent and treat carcinoma cancer. As described above, TGF‐β‐stimulated TβR‐I–TβR‐II‐mediated canonical signaling is responsible for potentiating TGF‐β growth inhibition mediated by TβR‐V by transcriptional activation of CDK inhibitors which activates the TβR‐V‐mediated tumor suppressor signaling cascade (TβR‐V/IRS‐2/PP1/pRb) to maintain pRb unphosphorylated (active). Small molecule TGF‐β enhancers (statins, vitamin D2, vitamin D3, cyanidin, apocyanin, dynasore, resveratrol, aspirin, ethanol, and DMSO), which enhance TGF‐β activity in epithelial cells, have been identified using a TGF‐β‐stimulated luciferase reporter gene assay in MLE cells‐Clone 32.^69^, ^72^, ^73^, ^74^, ^75^, ^76^ These small molecule enhancers enhance TGF‐β‐stimulated TβR‐I–TβR‐II‐mediated canonical signaling (TβR‐I/TβR‐II/Smad2/3/4) by recruiting TβR‐I–TβR‐II hetero‐oligomeric complexes from lipid rafts to non‐lipid raft microdomains^69^, ^72^, ^73^, ^75^, ^76^ and facilitating TGF‐β‐induced signaling at coated‐pit stages during clathrin‐mediated endocytosis.^74^ In epithelial cells, TβR‐V is mainly localized in plasma‐membrane non‐lipid raft microdomains which serve as signaling platforms for TGF‐β receptor (TβR‐I/TβR‐II/TβR‐V)‐mediated growth inhibition signaling. These TGF‐β enhancers could be used to prevent and treat the majority (carcinoma) of human cancers,^69^ and other chronic inflammatory diseases such as atherosclerotic cardiovascular disease (ASCVD).^77^, ^78^ Diets containing natural TGF‐β enhancers (fruits/vegetables/nuts rich in triterpenoids, polyphenols and antioxidants), synthetic TGF‐β enhancers (such as statins, resveratrol, cyanidin and aspirin), moderate ingestion of red wine, and exercise (which increases plasma HDL levels) are known to be associated with low risk of developing chronic inflammatory diseases such as cancer and ASCVD.^69^, ^73^, ^77^ HDL (high‐density lipoproteins), and ethanol enhance TGF‐β activity by recruiting TβR‐I and TβR‐II from cytoplasmic vesicles (intracellular pool) and lipid rafts/caveolae to non‐lipid raft microdomains.^73^, ^75^, ^78^ TGF‐β has been known to be a protective cytokine against carcinoma and ASCVD.^19^, ^79^

## CONFLICT OF INTEREST

The authors declare that there are no competing financial interest associated with this work.

## AUTHORS' CONTRIBUTIONS

C.L.C. performed most of the experiments in the Department of Biochemistry and Molecular Biology, Saint Louis University School of Medicine. F.W.H. was involved in identifying the negative regulation of non‐Smad pathways by TβR‐V. S.S.H and J.S.H. were involved in research designing and finalizing the manuscript.

## References

[1] Baxter RC. IGF binding proteins in cancer: mechanistic and clinical insights. Nat Rev Cancer. 2014;14:329‐341. 24722429 10.1038/nrc3720

[2] O'Grady P, Huang SS, Huang JS. Expression of a new type high molecular weight receptor (type V receptor) of transforming growth factor β in normal and transformed cells. Biochem Biophys Res Commun. 1991;179:378‐385. 1652955 10.1016/0006-291x(91)91381-l

[3] O'Grady P, Kuo MD, Baldassare JJ, Huang SS, Huang JS. Purification of a new type high molecular weight receptor (type V receptor) of transforming growth factor β (TGF‐β) from bovine liver. Identification of the type V TGF‐β receptor in cultured cells. J Biol Chem. 1991;266:8583‐8589. 1850748

[4] Leal SM, Liu Q, Huang SS, Huang JS. The type V transforming growth factor β receptor is the putative insulin‐like growth factor‐binding protein 3 receptor. J Biol Chem. 1997;272:20572‐20576. 9252371 10.1074/jbc.272.33.20572

[5] Leal SM, Huang SS, Huang JS. Interactions of high affinity insulin‐like growth factor‐binding proteins with the type V transforming growth factor‐β receptor in mink lung epithelial cells. J Biol Chem. 1999;274:6711‐6717. 10037769 10.1074/jbc.274.10.6711

[6] Leal SM. Characterization of the interactions of IGFBP‐3 with the type V TGF‐β receptor. Ph.D. dissertation, 1999; Saint Louis University, St. Louis, Missouri, USA.

[7] Huang SS, Huang JS. TGF‐β control of cell proliferation (Review). J Cell Biochem. 2005;96:447‐462. 16088940 10.1002/jcb.20558

[8] Herz J, Hamann U, Rogne S, Myklebost O, Gausepohl H, Stanley KK. Surface location and high affinity for calcium of a 500‐kd liver membrane protein closely related to the LDL‐receptor suggest a physiological role as lipoprotein receptor. EMBO J. 1988;7:4119‐4127. 3266596 10.1002/j.1460-2075.1988.tb03306.x PMC455121

[9] Liu Q, Huang SS, Huang JS. Function of the type V transforming growth factor β receptor in transforming growth factor β‐induced growth inhibition of mink lung epithelial cells. J Biol Chem. 1997;272:18891‐18895. 9228067 10.1074/jbc.272.30.18891

[10] Huang SS, Ling TY, Tseng WF, et al. Cellular growth inhibition by IGFBP‐3 and TGF‐β1 requires LRP‐1. FASEB J. 2003;17:2068‐2089. 14597676 10.1096/fj.03-0256com

[11] Huang SS, Liu Q, Johnson FE, Konish Y, Huang JS. Transforming growth factor‐β peptide antagonists and their conversion to partial agonists. J Biol Chem. 1997;272:27155‐27159. 9341157 10.1074/jbc.272.43.27155

[12] Huang SS, Zhou M, Johnson FE, Hsieh H‐S, Huang JS. An active site of transforming growth factor‐β_1_ for growth inhibition and stimulation. J. Biol. Chem. 1999;274:27754‐27758. 10488119 10.1074/jbc.274.39.27754

[13] Heldin CH, Miyazono K, ten Dijke P. TGF‐β signaling from cell membrane to nucleus through SMAD proteins. Nature. 1997;390:465‐471. 9393997 10.1038/37284

[14] Reynisdóttir I, Polyak K, Iavarone A, Massagué J. Kip/Cip and Ink4 Cdk inhibitors cooperate to induce cell cycle arrest in response to TGF‐β. Genes Dev. 1995;9:1831‐1845. 7649471 10.1101/gad.9.15.1831

[15] Tseng WF, Huang SS, Huang JS. LRP‐1/TβR‐V mediates TGF‐β1‐induced growth inhibition in CHO cells. FEBS Lett. 2004;562:71‐78. 15044004 10.1016/S0014-5793(04)00185-1

[16] Huang XY, Shi GM, Devbhandari RP, et al. Low level of low‐density lipoprotein receptor‐related protein 1 predicts an unfavorable prognosis of hepatocellular carcinoma after curative resection. PLoS One. 2012;7:e32775. 22427881 10.1371/journal.pone.0032775 PMC3299691

[17] Boulagnon‐Rombi C, Schneider C, Leandri C, et al. LRP1 expression in colon cancer predicts clinical outcome. Oncotarget. 2018;9:8849‐8869. 29507659 10.18632/oncotarget.24225 PMC5823651

[18] Gilardoni MB, Remedi MM, Oviedo M, et al. Differential expression of Low density lipoprotein receptor‐related Protein 1 (LRP‐1) and matrix metalloproteinase‐9 (MMP‐9) in prostate gland: from normal to malignant lesions. Pathol Res Pract. 2017;213:66‐71. 27931798 10.1016/j.prp.2016.11.008

[19] Derynck R, Akhurst RJ, Balmain A. TGF‐β signaling in tumor suppression and cancer progression. Nat Genet. 2001;29:117‐129. 11586292 10.1038/ng1001-117

[20] Tomii K, Tsukuda K, Toyooka S, et al. Aberrant promoter methylation of insulin‐like growth factor binding protein‐3 gene in human cancers. Int J Cancer. 2007;120:566‐573. 17096329 10.1002/ijc.22341

[21] Roberts AB, Wakefield LM. The two faces of transforming growth factor β in carcinogenesis. Proc Natl Acad Sci USA. 2003;100:8621‐8623. 12861075 10.1073/pnas.1633291100 PMC166359

[22] Huang SS, Leal SM, Chen CL, Liu IH, Huang JS. Identification of insulin receptor substrate proteins as key molecules for the TβR‐V/LRP‐1‐mediated growth inhibitory signaling cascade in epithelial and myeloid cells. FASEB J. 2004;18:1719‐1721.FASEB J express article 10.1096/fj.04-1872fje. Published online Sept. 15, 2004a, page 1‐26. 15371331

[23] Huang SS, Leal SM, Chen CL, Liu IH, Huang JS. Cellular growth inhibition by TGF‐β1 involves IRS proteins. FEBS Lett. 2004;565:117‐121. 15135063 10.1016/j.febslet.2004.03.082

[24] Fitzgerald DJ, Fryling CM, Zanovsky A, et al. Pseudomonas exotoxin‐mediated selection yields cells with altered expression of low‐density lipoprotein receptor‐related protein. J Cell Biol. 1995;129:1533‐1541. 7790352 10.1083/jcb.129.6.1533 PMC2291175

[25] Herz J, Goldstein JL, Strickland DK, Ho YK, Brown MS. 39‐kDa protein modulates binding of ligands to low density lipoprotein receptor‐related protein/α2‐macroglobulin receptor. J Biol Chem. 1991;266:21232‐21238. 1718973

[26] Cohen P, Klumpp S, Schelling DL. An improved procedure for identifying and quantitating protein phosphatases in mammalian tissues. FEBS Lett. 1989;250:596‐600. 2546812 10.1016/0014-5793(89)80803-8

[27] Takai A, Eto M, Hirano K, Takeya K, Wakimoto T, Watanabe M. Protein phosphatases 1 and 2A and their naturally occurring inhibitors: current topics in smooth muscle physiology and chemical biology. J Physiol Sci. 2018;68:1‐17. 28681362 10.1007/s12576-017-0556-6 PMC5754374

[28] Gruppuso PA, Mikumo R, Brautigan DL. Braun L Growth arrest induced by transforming growth factor β1 is accompanied by protein phosphatase activation in human keratinocytes. J Biol Chem. 1991;266:3444‐3448. 1847373

[29] Laiho M, DeCaprio JA, Ludlow JW, Livingston DM, Massagué J. Growth inhibition by TGF‐β linked to suppression of retinoblastoma protein phosphorylation. Cell. 1990;62:175‐185. 2163767 10.1016/0092-8674(90)90251-9

[30] Chao R, Khan W, Hannun YA. Retinoblastoma protein dephosphorylation induced by D‐erythro‐sphingosine. J Biol Chem. 1992;267:23459‐23462. 1385423

[31] Egger JV, Lane MV, Antonucci LA, Dedi B, Krucher NA. Dephosphorylation of the Retinoblastoma protein (Rb) inhibits cancer cell EMT via Zeb. Cancer Biol Ther. 2016;17:1197‐1205. 27645778 10.1080/15384047.2016.1235668 PMC5137485

[32] Mittnacht S, Weinberg RA. G1/S phosphorylation of the retinoblastoma protein is associated with an altered affinity for the nuclear compartment. Cell. 1991;65:381‐393. 2018973 10.1016/0092-8674(91)90456-9

[33] Hertz EPT, Kruse T, Davey NE, et al. A conserved motif provides binding specificity to the PP2A‐B56 phosphatase. Mol Cell. 2016;63:686‐695. 27453045 10.1016/j.molcel.2016.06.024

[34] Garcia A, Cayla X, Caudron B, Deveaud E, Roncal F, Rebollo A. New insights in protein phosphorylation: a signature for protein phosphatase 1 interacting proteins. C R Biol. 2004;327:93‐97. 15060979 10.1016/j.crvi.2004.01.001

[35] Sun H, Tu X, Prisco M, Wu A, Casiburi I, Baserga R. Insulin‐like growth factor 1 receptor signaling and nuclear translocation of insulin receptor substrates 1 and 2. Mol Endocrinol. 2003;17:472‐486. 12554758 10.1210/me.2002-0276

[36] Chen X, Ferry RJ Jr. Novel actions of IGFBP‐3 on intracellular signaling pathways of insulin‐secreting cells. Growth Horm IGF Res. 2006;16:41‐48. 16275148 10.1016/j.ghir.2005.09.003 PMC3092594

[37] Itoh S, Thorikay M, Kowanetz M, et al. Elucidation of Smad requirement in transforming growth factor‐β type I receptor‐induced responses. J Biol Chem. 2003;278:3751‐3761. 12446693 10.1074/jbc.M208258200

[38] Sorrentino A, Thakur N, Grimsby S, et al. The type I TGF‐β receptor engages TRAF6 to activate TAK1 in a receptor kinase‐independent manner. Nat Cell Biol. 2008;10:1199‐1207. 18758450 10.1038/ncb1780

[39] Hamidi A, Song J, Thakur N, et al. TGF‐β promotes PI3K‐AKT signaling and prostate cancer cell migration through the TRAF6‐mediated ubiquitylation of p85α. Sci Signal. 2017:10;eaal4186. 28676490 10.1126/scisignal.aal4186

[40] Fleming YM, Ferguson GJ, Spender LC, et al. TGF‐β‐mediated activation of RhoA signalling is required for efficient (V12)HaRas and (V600E)BRAF transformation. Oncogene. 2009;28:983‐993. 19079344 10.1038/onc.2008.449

[41] Griswold‐Prenner I, Kamibayashi C, Maruoka EM, Mumby MC, Derynck R. Physical and functional interactions between type I transforming growth factor β receptors and Bα, a WD‐40 repeat subunit of phosphatase 2A. Mol Cell Biol. 1998;18:6595‐6604. 9774674 10.1128/mcb.18.11.6595 PMC109244

[42] Yu N, Kozlowski JM, Park II, et al. Overexpression of transforming growth factor β1 in malignant prostate cells is partly caused by a runaway of TGF‐β1 auto‐induction mediated through a defective recruitment of protein phosphatase 2A by TGF‐β type I receptor. Urol. 2010;76(1519):e8‐13. 10.1016/j.urology.2010.03.061 PMC2997920 21030067

[43] Zhao L, He LR, Zhang R, et al. Low expression of IGFBP‐3 predicts poor prognosis in patients with esophageal squamous cell carcinoma. Med Oncol. 2012;29:2669‐2676. 22167391 10.1007/s12032-011-0133-4

[44] Yan J, Yang X, Li L, et al. Low expression levels of insulin‐like growth factor binding protein‐3 are correlated with poor prognosis for patients with hepatocellular carcinoma. Oncol Lett. 2017;13:3395‐3402. 28521445 10.3892/ol.2017.5934 PMC5431398

[45] Torng PL, Lin CW, Chan MW, Yang HW, Huang SC, Lin CT. Promoter methylation of IGFBP‐3 and p53 expression in ovarian endometrioid carcinoma. Mol Cancer. 2009;8:120. 20003326 10.1186/1476-4598-8-120 PMC2799391

[46] Lambrecht C, Libbrecht L, Sagaert X, et al. Loss of protein phosphatase 2A regulatory subunit B56δ promotes spontaneous tumorigenesis *in vivo* . Oncogene. 2018;37:544‐552. 28967903 10.1038/onc.2017.350

[47] Herz J, Clouthier DE, Hammer RE. LDL receptor‐related protein internalizes and degrades uPA‐PAI‐1 complexes and is essential for embryo implantation. Cell. 1992;71:411‐421. 1423604 10.1016/0092-8674(92)90511-a

[48] Lin SC, Skapek SX, Lee EY. Genes in the RB pathway and their knockout in mice. Semin Cancer Biol. 1996;7:279‐289. 9110405 10.1006/scbi.1996.0036

[49] Ingermann AR, Yang YF, Han J, et al. Identification of a novel cell death receptor mediating IGFBP‐3‐induced anti‐tumor effects in breast and prostate cancer. J Biol Chem. 2010;285:30233‐30246. 20353938 10.1074/jbc.M110.122226 PMC2943278

[50] Cai Q, Dozmorov M, Oh Y. IGFBP‐3/IGFBP‐3 receptor system as an anti‐tumor and anti‐metastatic signaling in cancer. Cells. 2020;9:1261. 10.3390/cells9051261 PMC7290346 32443727

[51] Joyce S, Nour AM. Blocking transmembrane 219 protein signaling inhibits autophagy and restores normal cell death. PLoS One. 2019;14:e0218091. 31220095 10.1371/journal.pone.0218091 PMC6586287

[52] Howe PH, Cunningham MR, Leof EB. Distinct pathways regulate transforming growth factor β1‐stimulated proto‐oncogene and extracellular matrix gene expression. J Cell Physiol. 1990;42:39‐45. 10.1002/jcp.1041420106 2153688

[53] Chen RH, Miettinen PJ, Maruoka EM, Choy L, Derynck R. A WD‐domain protein that is associated with and phosphorylated by the type II TGF‐β receptor. Nature. 1995;377:548‐552. 7566156 10.1038/377548a0

[54] Macias‐Silva M, Abdollah S, Hoodless PA, Pirone R, Attisano L, Wrana JL. MADR2 is a substrate of the TGF‐β receptor and its phosphorylation is required for nuclear accumulation and signaling. Cell. 1996;87:1215‐1224. 8980228 10.1016/s0092-8674(00)81817-6

[55] Hall FL, Benya PD, Padilla SR, et al. Transforming growth factor‐β type‐ll receptor signalling: intrinsic/associated casein kinase activity, receptor interactions and functional effects of blocking antibodies. Biochem J. 1996;316:303‐310. 8645222 10.1042/bj3160303 PMC1217339

[56] Hocevar BA, Howe PH. Mechanisms of TGF‐β‐induced cell cycle arrest. Miner Electrolyte Metab. 1998;24:131‐135. 9525695 10.1159/000057360

[57] Heldin NE, Bergstrom D, Hermansson A, et al. Lack of responsiveness to TGF‐β in a thyroid carcinoma cell line with functional type I and type II TGF‐β receptors and Smad proteins, suggests a novel mechanism for TGF‐β insensitivity in carcinoma cells. Mol Cell Endocrinol. 1999;153:79‐90. 10459856 10.1016/s0303-7207(99)00086-6

[58] Fink SP, Swinler SE, Lutterbaugh JD, et al. Transforming growth factor‐β‐induced growth inhibition in a Smad4 mutant colon adenoma cell line. Cancer Res. 2001;61:256‐260. 11196171

[59] Petritsch C, Beug H, Balmain A, Oft M. TGF‐β inhibits p70 S6 kinase via protein phosphatase 2A to induce G(1) arrest. Genes Dev. 2000;14:3093‐3101. 11124802 10.1101/gad.854200 PMC317138

[60] Moulder KM. Role of Ras and Mapks in TGF‐β signaling. Cytokine Growth Factor Rev. 2000;11:23‐35. 10708950 10.1016/s1359-6101(99)00026-x

[61] Stenvers KL, Tursky ML, Harder KW, et al. Heart and liver defects and reduced transforming growth factor β2 sensitivity in transforming growth factor β type III receptor‐deficient embryos. Mol Cell Biol. 2003;23:4371‐4385. 12773577 10.1128/MCB.23.12.4371-4385.2003 PMC156130

[62] Benes P, Jurajda M, Zaloudík J, Izakovicová‐Hollá L, Vácha J. C766T low‐density lipoprotein receptor‐related protein 1 (LRP1) gene polymorphism and susceptibility to breast cancer. Breast Cancer Res. 2003;5:R77‐81. 12793904 10.1186/bcr591 PMC165006

[63] Westermarck J, Li SP, Kallunki T, Han J, Kähäri VM. p38 mitogen‐activated protein kinase‐dependent activation of protein phosphatases 1 and 2A inhibits MEK1 and MEK2 activity and collagenase 1 (MMP‐1) gene expression. Mol Cell Biol. 2001;21:2373‐2383. 11259586 10.1128/MCB.21.7.2373-2383.2001 PMC86870

[64] Grallert A, Boke E, Hagting A, et al. A PP1‐PP2A phosphatase relay controls mitotic progression. Nature. 2015;517:94‐98. 25487150 10.1038/nature14019 PMC4338534

[65] Razani B, Zhang XL, Bitzer M, von Gersdorff G, Bottinger EP, Lisanti MP. Caveolin‐1 regulates transforming growth factor (TGF)‐β/SMAD signaling through an interaction with the TGF‐β type I receptor. J Biol Chem. 2001;276:6727‐6738. 11102446 10.1074/jbc.M008340200

[66] Zuo W, Chen YG. Specific activation of mitogen‐activated protein kinase by transforming growth factor‐β receptors in lipid rafts is required for epithelial cell plasticity. Mol Biol Cell. 2009;20:1020‐1029. 19056678 10.1091/mbc.E08-09-0898 PMC2633387

[67] McClinch K, Avelar RA, Callejas D, et al. Small‐molecule activators of protein phosphatase 2A for the treatment of castration‐resistant prostate cancer. Cancer Res. 2018;78:2065‐2080. 29358171 10.1158/0008-5472.CAN-17-0123 PMC5899650

[68] Hannigan A, Smith P, Kalna G, et al. Epigenetic downregulation of human disabled homolog 2 switches TGF‐β from a tumor suppressor to a tumor promoter. J Clin Invest. 2010;120:2842‐2857. 20592473 10.1172/JCI36125 PMC2912175

[69] Huang JS. Invited Faculty Speaker . Mechanism of switching TG‐β from a tumor suppressor to a tumor promoter: implications for the prevention and treatment of human cancer. *The 8th International Congress on Cancer metastasis*, San Francisco, CA, USA; 2019. Abstract:AB 22.

[70] Pampusch MS, Kamanga‐Sollo E, Hathaway MR, White ME, Dayton WR. Low‐density lipoprotein‐related receptor protein 1 (LRP‐1) is not required for insulin‐like growth factor binding protein 3 (IGFBP‐3) to suppress L6 myogenic cell proliferation. Domest Anim Endocrinol. 2011;40:197‐204. 21353438 10.1016/j.domaniend.2011.01.001

[71] Lee Y‐R, Chen M, Lee JD, et al. Reactivation of PTEN tumor suppressor for cancer treatment through inhibition of a MYC‐WWP1 inhibitory pathway. Science. 2019;364(6441):eaau0159. 31097636 10.1126/science.aau0159 PMC7081834

[72] Huang SS, Liu I‐H, Chen C‐L, Chang JM, Johnson FE, Huang JS. 7‐Dehydrocholesterol (7‐DHC), but not cholesterol, causes suppression of canonical TGF‐β signaling and is likely involved in the development of atherosclerotic cardiovascular disease (ASCVD). J Cell Biochem. 2017;118:1387‐1400. 27862220 10.1002/jcb.25797 PMC6123222

[73] Huang JS, Huang FW, Johnson FE, Huang SS. Molecular basis of diets, exercise and lifestyle interventions in atherosclerotic cardiovascular disease. In: Lewis BS, Borer JS, Halon DA, eds. The Proceedings of the 11th International Congress on Coronary Artery Disease, 2015:49‐52.

[74] Chen C‐L, Hou W‐H, Liu I‐H, Huang SS, Huang JS. Inhibitors of clarthrin‐dependent endocytosis inhibitors enhance TGF‐β signaling and responses. J Cell Sci. 2009;122:1863‐1871. 19461075 10.1242/jcs.038729 PMC2684837

[75] Huang SS, Chen CL, Huang FW, Johnson FE, Huang JS. Ethanol enhances TGF‐β Activity by recruiting TGF‐β receptors from intracellular vesicles/lipid rafts/caveolae to non‐lipid raft microdomains. J Cell Biochem. 2016;117:860‐871. 26419316 10.1002/jcb.25389 PMC6123223

[76] Huang SS, Chen CL, Huang FW, Hou W‐H, Huang JS. Dimethyl sulfoxide enhances TGF‐β activity by recruiting the type II TGF‐β receptor from intracellular vesicles/lipid rafts/caveolae to the plasma membrane. J Cell Biochem. 2016;117:1568‐1579. 26587792 10.1002/jcb.25448 PMC6123219

[77] Arranz S, Chiva‐Blanch G, Valderas‐Martínez P, Medina‐Remón A, Lamuela‐Raventós RM, Estruch R. Wine, beer, alcohol and polyphenols on cardiovascular disease and cancer. Nutrients. 2012;4:759‐781. 22852062 10.3390/nu4070759 PMC3407993

[78] Mahdy Ali K, Wonnerth A, Huber K, Wojta J. Cardiovascular disease risk reduction by raising HDL cholesterol – current therapies and future opportunities. Br J Pharmacol. 2012;167:1177‐1194. 22725625 10.1111/j.1476-5381.2012.02081.x PMC3504986

[79] Grainger DJ. Transforming growth factor and atherosclerosis: so far, so good for the protective cytokine hypothesis. Arterioscler Thromb Vasc Biol. 2004;24:399‐404. 14699019 10.1161/01.ATV.0000114567.76772.33

[80] Wu L, Derynck R. Essential role of TGF‐β signaling in glucose‐induced cell hypertrophy. Dev Cell. 2009;17:35‐48. 19619490 10.1016/j.devcel.2009.05.010 PMC2722039

